# Cx31.1 can selectively intermix with co-expressed connexins to facilitate its assembly into gap junctions

**DOI:** 10.1242/jcs.261631

**Published:** 2024-04-17

**Authors:** Stephanie E. Leighton, Robert S. Wong, Sergiu A. Lucaciu, Alexandra Hauser, Danielle Johnston, Peter B. Stathopulos, Donglin Bai, Silvia Penuela, Dale W. Laird

**Affiliations:** ^1^ Department of Anatomy and Cell Biology, University of Western Ontario, London, ON N6A 5C1, Canada; ^2^ Department of Physiology and Pharmacology, University of Western Ontario, London, ON N6A 5C1, Canada; ^3^ Western's Bone and Joint Institute, The Dr. Sandy Kirkley Centre for Musculoskeletal Research, University Hospital, London, ON N6A 5B9, Canada; ^4^ Division of Experimental Oncology, Department of Oncology, University of Western Ontario, London, ON N6A 5W9, Canada

**Keywords:** Connexin, Gap junction, Gap junctional intercellular communication, Cx31.1, Trafficking, Keratinocyte

## Abstract

Connexins are channel-forming proteins that function to facilitate gap junctional intercellular communication. Here, we use dual cell voltage clamp and dye transfer studies to corroborate past findings showing that Cx31.1 (encoded by *GJB5*) is defective in gap junction channel formation, illustrating that Cx31.1 alone does not form functional gap junction channels in connexin-deficient mammalian cells. Rather Cx31.1 transiently localizes to the secretory pathway with a subpopulation reaching the cell surface, which is rarely seen in puncta reminiscent of gap junctions. Intracellular retained Cx31.1 was subject to degradation as Cx31.1 accumulated in the presence of proteasomal inhibition, had a faster turnover when Cx43 was present and ultimately reached lysosomes. Although intracellularly retained Cx31.1 was found to interact with Cx43, this interaction did not rescue its delivery to the cell surface. Conversely, the co-expression of Cx31 dramatically rescued the assembly of Cx31.1 into gap junctions where gap junction-mediated dye transfer was enhanced. Collectively, our results indicate that the localization and functional status of Cx31.1 is altered through selective interplay with co-expressed connexins, perhaps suggesting Cx31.1 is a key regulator of intercellular signaling in keratinocytes.

## Teaser

**Summary:** The co-expression of select connexin isoforms enhances the ability of Cx31.1 to form gap junctions in rat epidermal keratinocytes.

## INTRODUCTION

Gap junctions are specialized transmembrane channels composed of connexin proteins that canonically function to facilitate the direct intercellular exchange of small molecules, ions and metabolites in a process called gap junctional intercellular communication (GJIC) (Zhou et al., 2023; Laird and Lampe, 2022; Lucaciu et al., 2023b). The scope of potential transjunctional molecules involved in GJIC is enormous and includes common signaling molecules, such as ATP, AMP, inositol trisphosphate and Ca^2+^ (Leybaert et al., 2017; Mese et al., 2007; Harris, 2007). Connexins belong to a diverse 21-member protein family that oligomerize into homomeric or heteromeric connexons frequently referred to as hemichannels (Aasen et al., 2018). Once they reach the cell surface, hemichannels can function in paracrine signaling or dock with apposing hemichannels, forming a variety of homotypic or heterotypic channel configurations that cluster into large semicrystalline arrays (Aasen et al., 2019; Lauf et al., 2002; Peng et al., 2022).

Although cells generally express two or more different connexins, channel complexity is thought to be enhanced in the epidermis with human and rodent keratinocytes differentially expressing numerous distinct isoforms (Cx26, Cx30, Cx30.3, Cx31, Cx31.1, Cx32, Cx37, Cx40, Cx43, and Cx45; designated in the current study as ‘keratinocyte connexins’) (Di et al., 2001; Faniku et al., 2015; Goliger and Paul, 1994). Consequently, keratinocyte connexins oligomerize into homomeric or heteromeric connexons *en route* to the plasma membrane, where they proceed to form homotypic or heterotypic channels that can exhibit distinct permeabilities and biophysical properties, which uniquely contribute to the maintenance of epidermal homeostasis and physiological function through GJIC (Lucaciu et al., 2023b; Martin et al., 2014; Scott et al., 2012; Martin and van Steensel, 2015; Koval et al., 2014). However, several reports suggest that Cx31.1 (encoded by *GJB5*) fails to assemble into functional gap junction channels in *Xenopus* oocytes and HeLa cells, but it is not known whether this is the case in connexin-rich keratinocytes where Cx31.1 is endogenously expressed in mammals and, in some cases, seen as puncta at the cell membrane (Hennemann et al., 1992; Bruzzone et al., 1994; Manthey et al., 1999; Manthey et al., 2001; Harris, 2001; Nugent et al., 2021; Goliger and Paul, 1994; Di et al., 2001; Chang et al., 2009).

In mouse models, studies using a Cx31.1-deficient LacZ reporter mouse have demonstrated that Cx31.1 is a crucial connexin as ∼30% of Cx31.1-deficient mice die *in utero* due to defects in spongiotrophoblast and labyrinth layers of the placenta (Zheng-Fischhofer et al., 2007). Surviving mice exhibit β-galactosidase staining in the suprabasal layers of the epidermis, confirming epidermal expression of Cx31.1 (Zheng-Fischhofer et al., 2007). Although not studied in detail, the epidermal architecture of these mutant mice appeared to be normal, possibly implying that other keratinocyte connexins functionally compensate for the loss of Cx31.1 (Zheng-Fischhofer et al., 2007). Cx31.1 also appears to be regulated in rodent skin as Cx31.1 levels are reduced at the edge of rat tail wounds (Goliger and Paul, 1994). Conversely, Cx31.1 is elevated at scrape-wounded edges of HaCaT human keratinocyte cultures at 24 h following wounding (Goliger and Paul, 1994; Nugent et al., 2021). Cx31.1 might additionally have a role in skin cancer as regions of hyperplastic skin and papillomas exhibit reduced levels of Cx31.1 in various chemically induced mouse models of skin cancer (Budunova et al., 1996a,b). Similarly, Cx31.1 is significantly downregulated in human metastatic melanoma lesions and subsequent gene expression profile analysis have indicated that higher Cx31.1 levels are correlated with improved overall patient survival (Scatolini et al., 2022). This observation was previously supported when the ectopic expression of Cx31.1 significantly inhibited the malignant phenotype of non-small cell lung cancer cells *in vitro* and *in vivo* despite its apparent inability to participate in GJIC, raising questions about if, where, and when Cx31.1 participates in GJIC (Zhang et al., 2012).

In this study, we investigated the life cycle, functional status and crosstalk between Cx31.1 and other keratinocyte connexins in rat epidermal keratinocytes (REKs) and REKs where Cx43 (encoded by *Gja1*) was ablated to render REKs GJIC deficient. Our results support past findings that Cx31.1 does not form functional gap junctions, largely due to a trafficking defect that sees only small quantities reach the cell surface, with little evidence of cell surface puncta. Cx31.1 did not accumulate in any single intracellular secretory or degradation compartment but did increase in the presence of proteosome inhibition and colocalized to lysosomes where it is likely to be degraded. We also found that Cx31.1 interacted with intracellular pools of Cx43, which shortened its relative half-life. It was particularly striking that Cx31 or Cx26 co-expression drove Cx31.1 assembly into gap junctions where Cx31.1–Cx31 intermixed channels demonstrated enhanced GJIC.

## RESULTS

### Cx31.1 is intracellularly retained in rat epidermal keratinocytes

**Fig. 1.**
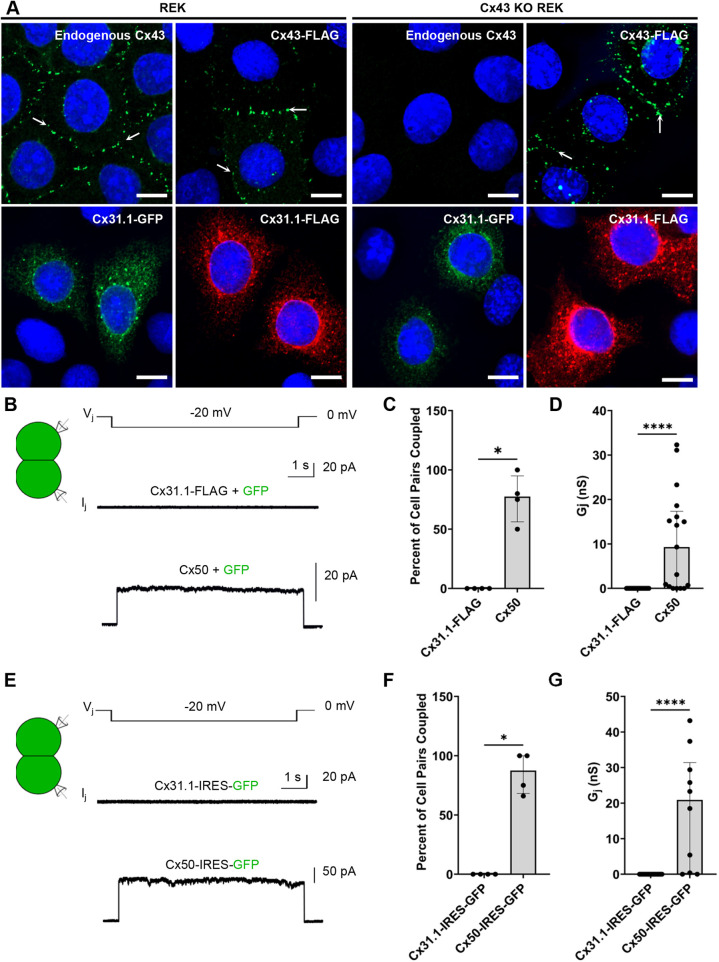
**Cx31.1 is intracellularly localized and fails to form functional gap junction channels.** (A) REKs and Cx43 KO REKs ectopically expressing Cx31.1–FLAG (red) or Cx31.1–GFP (green) failed to assemble Cx31.1 into gap junction plaques at cell–cell interfaces. In parallel studies, Cx43–FLAG (green) or endogenous Cx43 (green) formed gap junctions in REKs (arrows). Nuclei were stained with Hoechst 33342 (blue). Representative of *n*=3. Scale bars: 10 µm. (B,E) Representative transjunctional current (*I*_j_) in response to a −20 mV transjunctional voltage pulse (*V*_j_) in AD-293 cell pairs (with Cx43 and Cx45 ablated) engineered to co-express GFP in combination with Cx31.1–FLAG or mouse Cx50 (control) (*n*=4) (B) or engineered to express Cx31.1-IRES-GFP or Cx50-IRES-GFP (control) (*n*=3) (E). (C,D,F,G) Quantification of the median (with interquartile range) percentage of cell pairs coupled per transfection (C,F) and median with interquartile range coupling conductance (*G*_j_) of each individual cell pair (D,G) revealing that Cx31.1–FLAG and untagged Cx31.1-IRES-GFP do not form functional gap junction channels. *P<0.05; ****P<0.0001 (Mann–Whitney tests to compare coupling percentage and *G*_j_ between Cx31.1–FLAG and Cx50, and between Cx31.1-IRES-GFP and Cx50-IRES-GFP, respectively). Points in C and F represent the number of independent transfections; in D and G, points are cell pairs recorded, and in all cases error bars are interquartile range.

Phenotypically similar to basal keratinocytes, REKs endogenously express an abundant level of the gap junction forming protein Cx43 (Jakobsen et al., 2022; Maher et al., 2005). To investigate the subcellular localization and functional capacity of Cx31.1 in keratinocytes, we expressed FLAG- and GFP-tagged Cx31.1 in GJIC-competent Cx43-enriched and Cx43-ablated REKs (Cx43 KO). REKs engineered with an ablation of Cx43 were included in this study as they have been previously shown to be GJIC deficient (Au et al., 2020; Lucaciu et al., 2022). We observed that when FLAG- or GFP-tagged Cx31.1 were expressed in either REKs or Cx43 KO REKs, Cx31.1 rarely assembled into gap junction-like puncta at cell–cell interfaces unlike endogenous and FLAG-tagged Cx43 (Fig. 1A). Rather, both tagged versions of Cx31.1 were found predominantly within intracellular compartments regardless of the presence or absence of endogenous Cx43. Keratinocytes transfected (24–35% efficiency) with Cx31.1–FLAG did not show any apparent cell death (Fig. S1A,B) eliminating the possibility of any transfection-based toxicity.

To determine whether Cx31.1 could form functional gap junction channels that cannot be optically resolved by fluorescence microscopy, the functional capacity of Cx31.1 was assessed by dual whole-cell voltage clamp in AD-293 cells where Cx43 and Cx45 were ablated to render these cells GJIC deficient. The AD-293 GJIC-deficient cells were transiently transfected with untagged mouse Cx50 as a gap junction competent control or FLAG-tagged Cx31.1 at a 5:1 ratio with GFP to visually identify connexin-expressing cells. Dual whole-cell patch clamp electrophysiology was used to measure junctional currents (*I*_j_) in GFP-positive cell pairs following the application of a −20 mV voltage pulse (*V*_j_) (Fig. 1B–D). Cx31.1–FLAG-expressing cell pairs exhibited no *I*_j_s in response to the tested *V*_j_ unlike cell pairs expressing Cx50 (Fig. 1B–D). Quantification of the median percentage of cell pairs coupled per transfection and the coupling conductance (*G*_j_) of each individual cell pair revealed that Cx31.1–FLAG failed to form functional intercellular channels (Fig. 1B–D). Similarly, FLAG-tagged Cx31.1-expressing cell pairs displayed no *I*_j_s in response to a series of *V*_j_ pulses unlike Cx50-expressing cell pairs which displayed *I*_j_s showing expected *V*_j_-dependent gating properties (Fig. S2A). In parallel the functional status of untagged Cx31.1 was assessed by expressing Cx31.1-IRES-GFP (in a bicistronic vector) in GJIC-deficient AD-293 cells (Fig. 1E–G). In all experimental paradigms, Cx31.1 failed to form functional gap junction channels unlike Cx50, which formed functional channels in all situations tested (Fig. 1E–G; Fig. S2B).

### Minimal levels of Cx31.1 are found at the cell surface

**Fig. 2.**
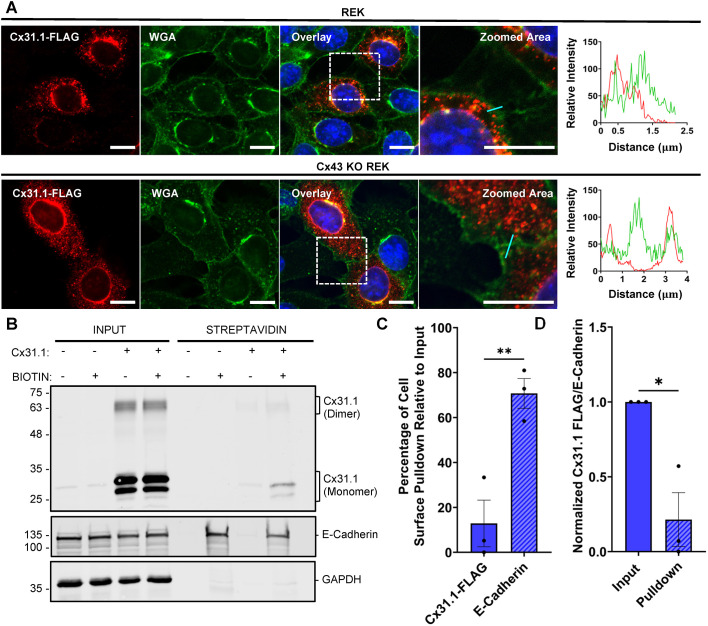
**Low levels of FLAG-tagged Cx31.1 are found at the cell surface.** (A) Cx31.1–FLAG (red) does not optically colocalize with cell surface WGA (green) in REKs or Cx43 KO REKs. Dashed boxes denote magnified areas. The fluorescent intensity profile of each representative image was quantified along the cyan line. Nuclei were stained with Hoechst 33342 (blue). Representative of *n*=3. Relative intensity is in arbitrary units. Scale bars: 10 µm. (B) Cell surface biotinylation assay immunoblot indicating that only a small fraction of Cx31.1–FLAG was localized to the surface of REKs. Input lysates were prepared in parallel with the absence of streptavidin pulldown. E-cadherin and GAPDH were used as positive and negative controls, respectively. (C,D) Quantification of Cx31.1–FLAG pulldown relative to biotinylated Cx31.1–FLAG input (C) and Cx31.1–FLAG relative to E-cadherin (D) indicating that low levels of cell surface Cx31.1–FLAG were detected. Error bars are mean±s.e.m.; *n*=3. *P≤0.05, **P≤0.01 (two-tailed unpaired Student's *t*-test).

Given that we found that Cx31.1 did not form functional intercellular channels, we next sought to uncover whether Cx31.1 was localized to the plasma membrane. FLAG-tagged Cx31.1-expressing REKs and Cx43 KO REKs exhibited minimal Cx31.1 colocalization with cell surface glycoproteins labeled with wheat germ agglutinin (WGA), a proxy marker to demarcate the location of the plasma membrane, which selectively binds N-acetylglucosamine and N-acetylneuraminic acid residues (Fig. 2A). To further interrogate whether Cx31.1 reached the cell surface, we performed cell surface biotinylation assays. These studies revealed that only a small subpopulation of Cx31.1–FLAG was available at the cell surface for biotinylation and subsequent pulldown with streptavidin beads (Fig. 2B). Typically, Cx31.1 presents as a doublet in western blots that we and others have seen, with considerable variability in the lower band suggesting a possible proteolytic cleavage (Zhu et al., 2015; Scatolini et al., 2022). Quantification of the cell surface pulldown relative to input or E-cadherin levels indicated that a small fraction of Cx31.1–FLAG was found on the surface of keratinocytes (Fig. 2C,D). To further interrogate whether a population of Cx31.1 could stably reach the cell surface, we performed time-lapse imaging of Cx43 KO REKs that expressed Cx31.1–GFP. In some, but not all cases, we found low levels of Cx31.1–GFP at the cell surface, with minimal evidence of gap junction-like structures, and this persisted for the duration of the imaging period (Fig. S3, boxed area). Collectively, these studies suggest that even though low levels of Cx31.1 reach the cell surface, this connexin is inefficient at assembling into gap junctions.

### Cx31.1 is found in many intracellular compartments

**Fig. 3.**
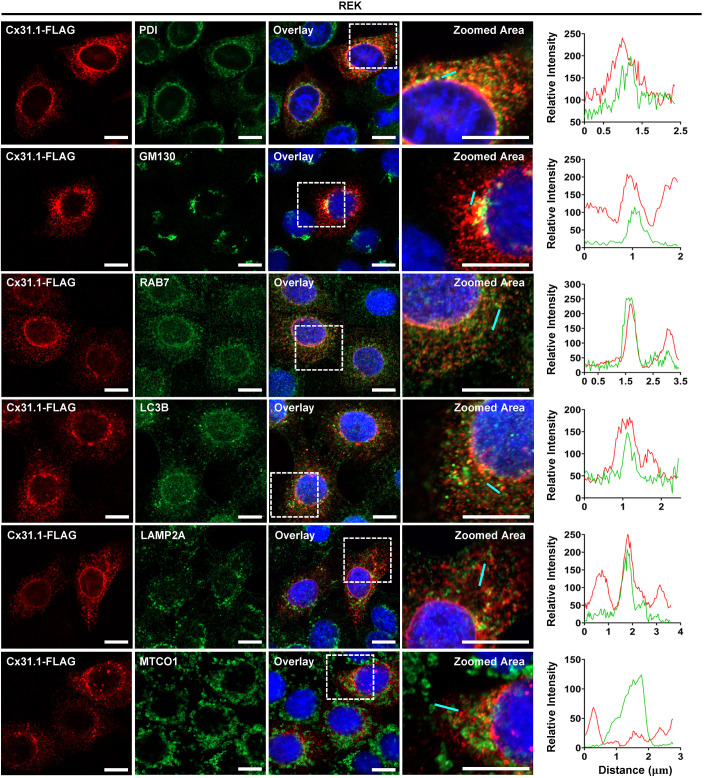
**Cx31.1 localizes to multiple intracellular compartments in REKs.** REKs expressing Cx31.1–FLAG (red) partially colocalized with resident proteins of the ER (green; anti-PDI antibody), Golgi (green; anti-GM130 antibody), late endosomes (green; anti-RAB7 antibody), autophagosomes (green; anti-LC3B antibody), and the lysosomes (green; anti-LAMP-2A antibody), suggesting Cx31.1–FLAG enters intracellular compartments linked to protein secretion and degradation. Cx31.1 failed to colocalize with mitochondria as denoted by the mitochondrial marker (green; anti-MTCO1 antibody). Dashed boxes denote zoomed imaged areas, and the fluorescent intensity profile of each representative image was quantified along the cyan line. Relative intensity is in arbitrary units. Nuclei are stained with Hoechst 33342 (blue). Representative of *n*=3. Scale bars: 10 µm.

To assess the intracellular compartments where Cx31.1 might be found in keratinocytes, REKs and Cx43 KO REKs expressing Cx31.1–FLAG were immunolabeled for FLAG as well as for protein disulfide isomerase (PDI), an endoplasmic reticulum (ER)-lumen resident protein, and Golgi matrix protein (GM130; also known as GOLGA2), a cis-Golgi marker. Cx31.1–FLAG was found to colocalize with both PDI and GM130; however, it did not substantially accumulate within either of these compartments (Fig. 3; Fig. S4). Even though Cx31.1 was found in the ER, this was insufficient to elevate the levels of binding immunoglobulin protein (BiP; also known as HSPA5), a marker of ER stress (Fig. S5). However, given that less than half of the REKs were transfected, we cannot rule out the possibility that high levels of Cx31.1 might elevate BiP expression. Thus, there was no indication by this one measure that Cx31.1 was causing ER stress as seen in tunicamycin-treated keratinocytes (Fig. S5A–C).

Given that the bulk of intracellular Cx31.1 did not accumulate within the ER or Golgi, we further investigated its potential localization to intracellular compartments en route to protein degradation and identified that Cx31.1–FLAG localized to autophagosomes (marked with microtubule-associated protein 1A/1B light chain 3β; LC3B, also known as MAP1LC3B), lysosomes (marked with lysosome-associated membrane glycoprotein 2; LAMP-2A), and late endosomes (marked with Ras-related protein Rab-7a; RAB7) (Fig. 3; Fig. S4). Cx31.1 was resolved in all these compartments suggesting it was being targeted for degradation. As might be expected, Cx31.1–FLAG did not notably localize to mitochondria as denoted by mitochondrially encoded cytochrome C oxidase I (MTCO1) labeling (Fig. 3; Fig. S4). Collectively, these studies suggest that Cx31.1 transiently localizes to, but does not accumulate in, any single intracellular compartment.

### Cx31.1 accumulates in the presence of a proteasomal inhibitor and redistributes with Cx43 when lysosomes are inhibited

**Fig. 4.**
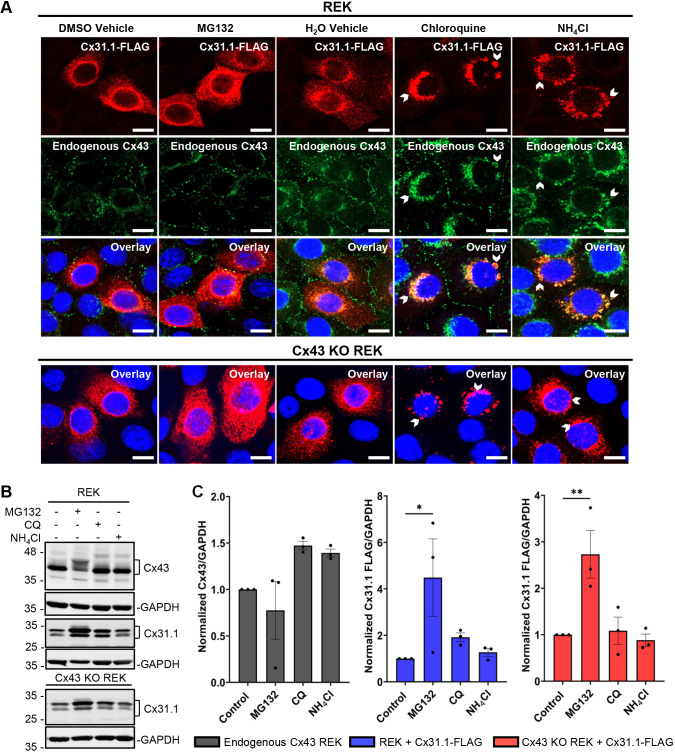
**Cx31.1 accumulates in the presence of a proteasomal inhibitor and relocalizes when lysosomes are inhibited.** (A) The immunofluorescence signal for Cx31.1–FLAG (red) appeared to be enhanced 4 h following MG132 (50 µM) inhibition of proteasomal function but not after NH_4_Cl (10 mM) or chloroquine (CQ; 100 µM) inhibition of lysosomal function. Lysosomal inhibitors increased the perinuclear localization of Cx31.1 and Cx43 (chevrons). Nuclei stained with Hoechst 33342 (blue). Representative of *n*=3. Scale bars: 10 μm. (B) Representative immunoblots of REKs and Cx43 KO REKs treated with MG132, NH_4_Cl or CQ for 10 h. (C) Quantification indicates that Cx31.1–FLAG levels are significantly enhanced following proteasomal inhibition. Results are mean±s.e.m., *n*=3. *P≤0.05, **P≤0.01 (one-way ANOVA with Dunnett's post test).

To assess the mechanisms that might govern Cx31.1 degradation we proceeded to inhibit proteasomal and lysosomal degradation. MG132 inhibition of proteasomes had no effect on the localization of Cx31.1 but enhanced Cx31.1-FLAG levels in REKs and Cx43 KO REKs with a preferential increase in the uppermost band found within the doublet of Cx31.1 (Fig. 4A–C). MG132 treatment had a minimal effect on Cx43 localization, but there was a significant change in the Cx43 banding patterns reflective of enhanced Cx43 phosphorylation (Banerjee et al., 2011; Solan and Lampe, 2020) (Fig. 4A–C). However, when lysosomal function was inhibited with ammonium chloride (NH_4_Cl) or chloroquine (CQ), which typically causes functionally impaired lysosomes to swell, Cx31.1 localization became more perinuclear and readily colocalized with endogenous Cx43. There was no apparent accumulation of Cx31.1–FLAG upon lysosomal inhibition (Misinzo et al., 2008; Mauthe et al., 2018).

### The relative half-life of Cx31.1 was reduced in Cx43-expressing keratinocytes

**Fig. 5.**
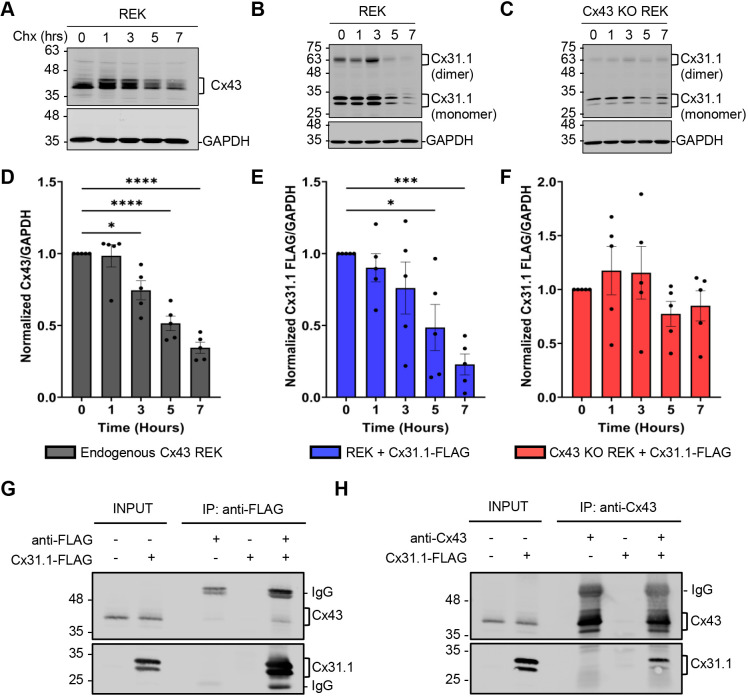
**Cx31.1 interacts with endogenous Cx43 increasing its relative rate of turnover.** Immunoblots of REKs and Cx43 KO REKs expressing Cx31.1–FLAG treated with cycloheximide (Chx; 10 µg/ml) and immunoblotted for (A) endogenous Cx43 or (B,C) ectopic Cx31.1–FLAG. (D–F) Quantification of Cx43 and Cx31.1–FLAG levels normalized to GAPDH. Cx31.1 was found to have a similar relative turnover to Cx43 when co-expressed and longer turnover when Cx43 was absent. Results are mean±s.e.m., *n*=5. *P≤0.05, ***P≤0.001; ****P≤0.0001 (one-way ANOVA with Dunnett's post test). (G,H) REKs expressing Cx31.1–FLAG were immunoprecipitated (IP) with anti-FLAG or anti-Cx43 antibody. Endogenous Cx43 was co-immunoprecipitated with anti-FLAG antibody and Cx31.1–FLAG with anti-Cx43 antibody suggesting these isoforms interact. Representative of *n*=3.

To determine the relative half-life of Cx31.1, REKs expressing Cx31.1 were treated with cycloheximide (Chx), a protein translation inhibitor, and the fate of Cx31.1 was assessed over time and compared to that of Cx43. Quantification of immunoblots revealed that exogenous Cx31.1–FLAG turnover paralleled that of endogenous Cx43 in wild-type REKs (Fig. 5A,B,D,E). Interestingly, in Cx43-ablated REKs, the turnover of Cx31.1 was delayed (Fig. 5C,F). To assess whether the increased rate of Cx31.1 turnover in wild-type REKs was due in part to a physical interaction with intracellular Cx43, we immunoprecipitated Cx31.1–FLAG and assessed whether endogenous Cx43 was co-immunoprecipitated. We found that a population of Cx31.1 co-immunoprecipitated Cx43 (Fig. 5G). Reciprocal immunoprecipitation of endogenous Cx43 similarly co-immunoprecipitated Cx31.1–FLAG (Fig. 5H). Although repeats of this analysis revealed that the beads only and isotype controls did occasionally exhibit low levels of non-specific binding to the protein G Dynabeads (Fig. S6), the levels were insufficient to negate the strong evidence that a population of Cx31.1 interacts with Cx43.

### Cx31 efficiently recruits Cx31.1 into gap junctions

**Fig. 6.**
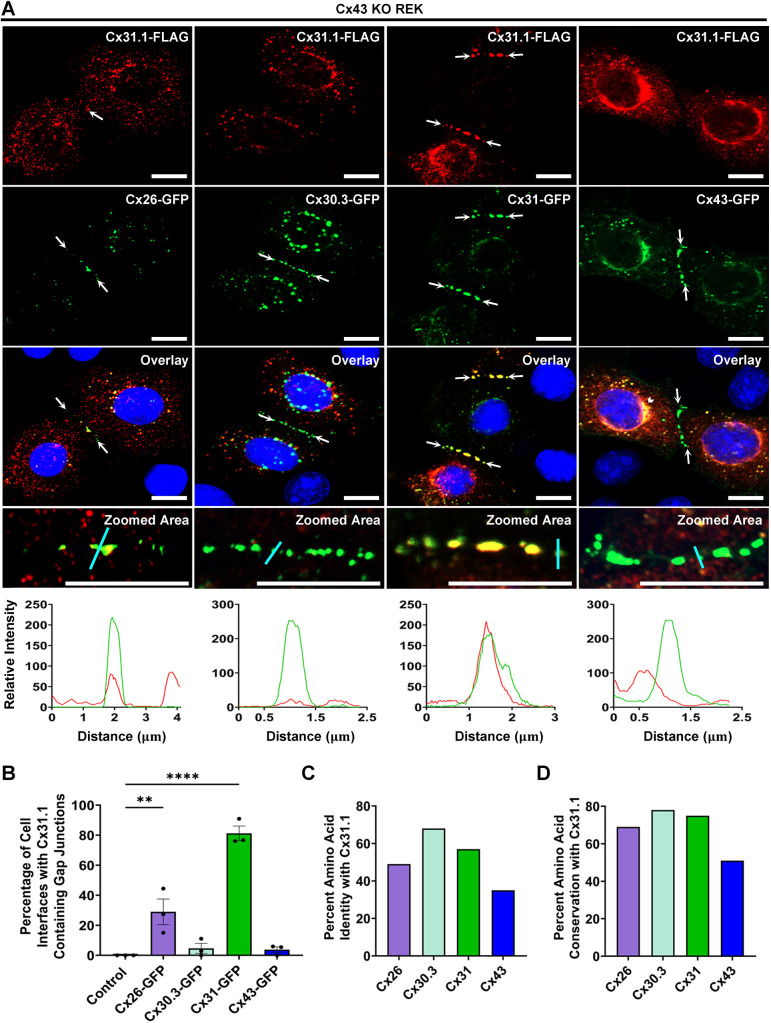
**Cx31 and Cx26 selectively recruit Cx31.1 into gap junctions.** (A,B) Co-expression of Cx30.3–GFP or Cx43–GFP was ineffective in driving Cx31.1–FLAG (red) assembly into gap junctions. Cx31–GFP, and to a much lesser extent Cx26–GFP, recruited Cx31.1–FLAG into the same gap junctions (arrows) suggesting they may intermix. The fluorescence intensity profile of each representative image was quantified along the cyan line. Nuclei stained with Hoechst 33342 (blue). Scale bars: 10 μm. Results are mean±s.e.m., *n*=3. **P≤0.01, ****P<0.0001 (one-way ANOVA with Dunnett's post test). (C,D) Basic local alignment search tool (BLAST) indicates that human Cx31.1 is most similar to the β-connexins Cx26, Cx30.3 and Cx31, as revealed by the higher percent connexin amino acid sequence identity and conservation (similarity) relative to the α-connexin Cx43.

Given that keratinocyte connexins are differentially expressed throughout the living layers of the epidermis, it is likely that Cx31.1 is spatially and temporally expressed in conjunction with one or more keratinocyte connexins. As such, we investigated whether Cx31.1 co-expression with select keratinocyte connexins could modulate its subcellular distribution. Co-expression with Cx30.3–GFP was ineffective in driving Cx31.1–FLAG assembly into gap junctions (Fig. 6A,B). Likewise, exogenous Cx43–GFP did not facilitate Cx31.1 gap junction assembly as expected based on the evidence that endogenous Cx43 did not recruit Cx31.1 into gap junctions. However, Cx31.1 and Cx43 appear to colocalize intracellularly, which is likely within a compartment where they interact. Interestingly, we found that the co-expression of Cx31.1–FLAG with Cx26–GFP resulted in a modest increase (29%) in Cx31.1 being found at the cell surface as gap junction plaques. However, Cx31–GFP efficiently recruited Cx31.1 into the same gap junctions (81%). It was particularly notable that Cx31.1 and Cx31 were evenly distributed within the same gap junctions (see Fig. 6A, zoomed image) suggesting they might intermix within the same connexons and form intercellular channels. Intriguingly, BlastP pairwise sequence alignment shows that Cx31 and Cx31.1 share high amino acid sequence identity and conservation (similarity) (Fig. 6C,D). Although Cx30.3 also shares high sequence identity with Cx31.1, it failed to recruit Cx31.1 into the same gap junctions suggesting there are further contributing factors that define what connexins will intermix. Overall, these findings suggest that the fate of Cx31.1 in keratinocytes is altered through interactions with select co-expressed connexins.

### Homology models predict Cx31.1 forms fewer intra- and inter-connexon hydrogen bonds and salt bridges than Cx31–Cx31.1 intermixed gap junctions

**Fig. 7.**
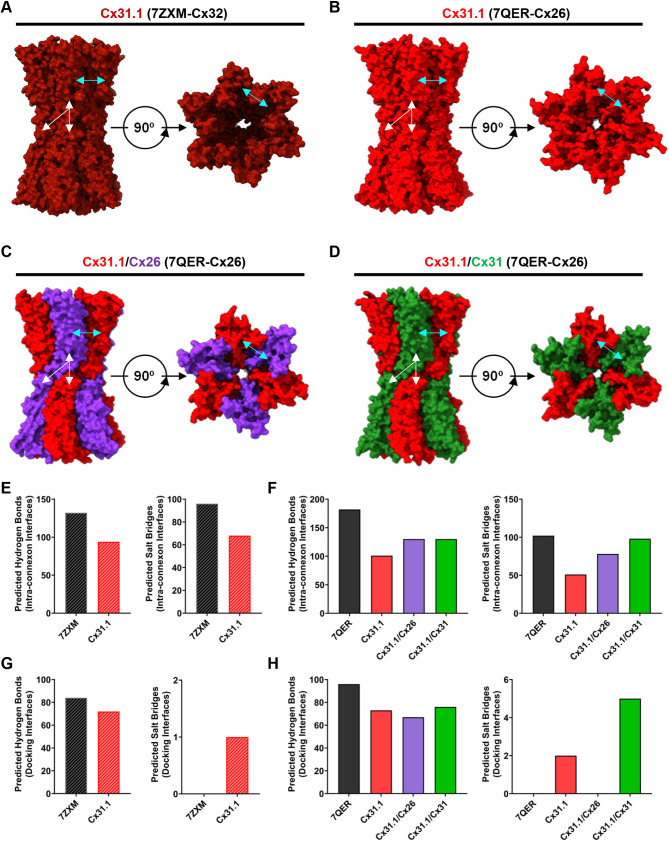
**Cx31.1 is predicted to form fewer intra- and inter-connexon hydrogen bonds and salt bridges overall than Cx31.1 intermixed channels.** Cx31.1 homomeric and homotypic homology model dodecamer based on the high-resolution structure of (A) Cx32 (PDB ID: 7ZXM) and (B) Cx26 (PDB ID: 7QER) depicted in surface view using Chimera X (Version 1.6.1). (C) Cx31.1–Cx26 and (D) Cx31.1–Cx31 intermixed gap junctions were generated based on the high-resolution structure of Cx26 (PDB ID: 7QER). Cyan arrows are utilized to denote intra-connexon interfaces and white arrows to indicate inter-connexon docking interfaces. (E,F) Intra-connexon hydrogen bond and salt bridge interactions for template and model structure reported by the PISA server. Homomeric and homotypic Cx31.1 homology models have fewer non-covalent (hydrogen bond and salt bridge) interactions at oligomer intra-connexon interfaces when compared to the corresponding template structures (see E,F). However, Cx31.1 intermixed channels are predicted to form more hydrogen bonds and salt bridges at intra-connexon interfaces compared to homomeric and homotypic Cx31.1 channels (see F). (G,H) Inter-connexon hydrogen and salt bridge interactions for template and model structures reported by the PISA server. Homomeric and homotypic Cx31.1 homology models are predicted to form fewer hydrogen bond interactions than the corresponding template structures (see G and H). However, the Cx31.1–Cx31 intermixed channels show a greater number of inter-connexon non-covalent interactions compared to homomeric and homotypic Cx31.1 channels. Values are a sum of all intra- or inter-connexon interfaces within the full gap junction channel.

To explore whether homotypic Cx31.1 fail to assemble into gap junctions due to channel structure instabilities, a homology Cx31.1 gap junction channel model was generated based on the high-resolution cryo-electron microscopy (cryoEM) structure of a homologous β-connexin, Cx32 (PDB ID: 7ZXM; Fig. 7A; Qi et al., 2023). The overall amino acid sequence identity and similarity between human Cx32 and Cx31.1 is 53% and 73%, respectively. Although not thought to be directly involved in connexin oligomerization or connexon docking, the complete cytoplasmic loop, N-terminal and C-terminal domain of Cx32 are not resolved and were not considered when analyzing potential interactions with the proteins, interfaces, structures and assemblies (PISA) server. Interestingly, PISA indicates that Cx31.1 forms fewer hydrogen bonds and salt bridges at intra-connexon interfaces when compared to template Cx32 (Fig. 7E). Although, the Cx31.1 homology model is predicted to have one additional salt bridge at the inter-connexon docking interfaces, Cx31.1 is predicted to form fewer hydrogen bonds (Fig. 7G). We also generated a second Cx31.1 homology model based on the cryoEM structure of Cx26 (PDB ID: 7QER; Fig. 7B; Brotherton et al., 2022). The overall amino acid sequence identity and similarity between human Cx26 and Cx31.1 is 49% and 69%, respectively. The homology model predicts Cx31.1 forms fewer hydrogen bonds and salts bridges compared to Cx26 at intra-connexon interfaces (Fig. 7F). Moreover, PISA indicates that the inter-connexon docking interfaces form fewer hydrogen bonds and more salt bridges when compared to Cx26 (Fig. 7H). Nevertheless, this overall reduction in non-covalent interactions might potentially impede Cx31.1 homo-oligomerization and docking capacity by lowering the stability between interfaces, thus contributing to GJIC incompetency.

As Cx31.1 was recruited into Cx31 and, to a lesser extent, Cx26 gap junctions, we similarly explored these heterotypic arrangements by homology modeling. Although the stoichiometry of isoforms within proposed Cx31.1–Cx31 and Cx31.1–Cx26 intermixed channels are undefined, and likely variable, we generated theoretical heteromeric heterotypic Cx31.1–Cx31 and Cx31.1–Cx26 gap junction channel models composed of both isoforms at a 1:1 ratio using the Cx26 cryoEM structure as the template (PDB ID: 7QER) (Fig. 7C,D). Interestingly, when considering the sum of all hydrogen bonds and salt bridges within all intra- and inter-connexon interfaces, Cx31.1–Cx31 and Cx31.1–Cx26 gap junction channels are predicted to form more non-covalent interactions when compared to the Cx31.1 homomeric homotypic homology model (Fig. 7F,H). Thus, increased non-covalent interactions in Cx31.1–Cx31 and Cx31.1–Cx26 might better support the assembly of these intermixed gap junction channels. We also note that Cx31.1 intermixed gap junction channel homology models are predicted to have an increased pore diameter compared to homomeric homotypic Cx31.1 gap junction channels (Fig. S7A–I). The pore diameter is narrowest where the N-terminal helix inserts into the pore, with the limitation that the cytoplasmic loop and C-terminus are not resolved. These differences in pore structure and ultimately the composition of pore lining residues might also contribute to differences in channel permeability and selectivity. Although, none of the predicted pore sizes are likely large enough to pass molecules such as ATP, the intermixed Cx31.1–Cx31 and Cx31.1–Cx26 gap junction channels would require smaller conformational changes to facilitate the passage of this type of molecule (Penkov and Penkova, 2021; Syrjanen et al., 2021).

### Co-expression of Cx31.1 with Cx31 enhances dye transfer

**Fig. 8.**
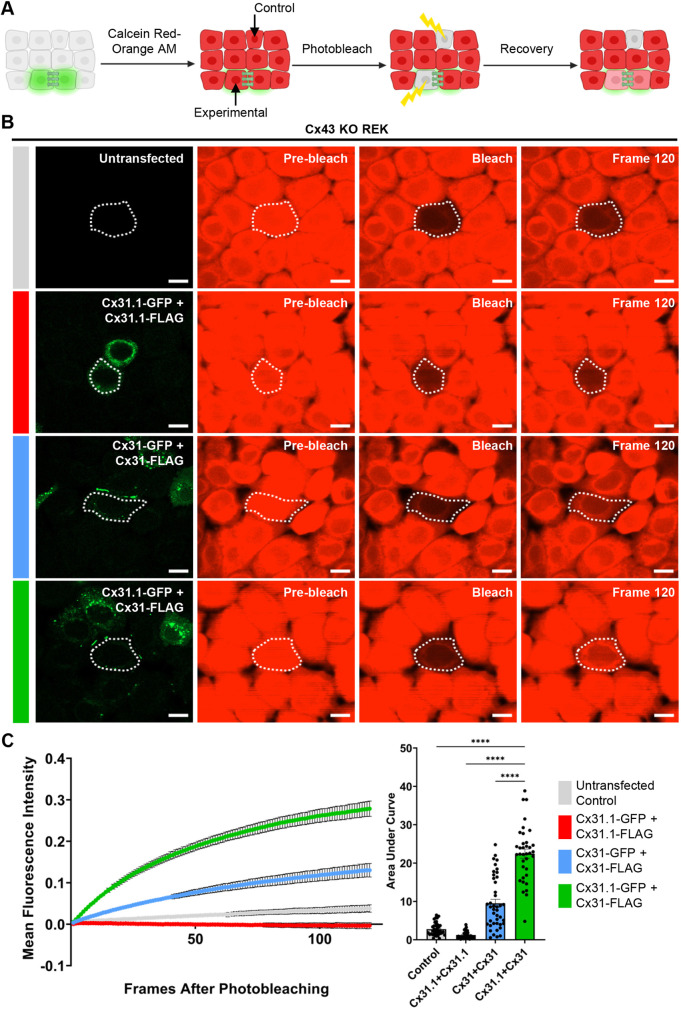
**Co-expression of Cx31.1 with Cx31 enhances gap junction channel-mediated dye transfer.** (A) Schematic of fluorescence recovery after photobleaching (FRAP) as a measure of GJIC. Created with BioRender. (B) Representative FRAP images of Cx43 KO REKs transfected with equal concentrations of plasmid DNA encoding Cx31.1–FLAG and Cx31.1–GFP (green), Cx31–FLAG and Cx31–GFP (green), or Cx31–FLAG and Cx31.1–GFP (green). Non-transfected Cx43 KO REKs were utilized as a baseline control. Photobleached areas are indicated with a dashed white line. Scale bars: 10 μm. (C) Mean±s.e.m. fluorescence recovery of Calcein Red–Orange dye transfer after photobleaching was assessed over 120 s (left). Quantification of the area (right, mean±s.e.m.) under the curve revealed that the co-expression of Cx31–FLAG with Cx31.1–GFP (green) significantly enhances GJIC. *N*=3 independent experiments with *n*=10–18 photobleached cells per biological replicate. ****P<0.0001 (one-way ANOVA with Dunnett's post test).

We next sought to determine whether Cx31.1–FLAG when assembled into gap junctions with Cx31 would alter Calcein Red-Orange dye transfer beyond what would be observed when Cx31 was expressed alone. Fluorescence recovery after photobleaching (FRAP) was recorded over 120 s in untransfected Cx43 KO REKs or Cx43 KO REKs expressing combinations of Cx31.1 and Cx31 (Fig. 8A,B). When Cx43 KO REKs were co-transfected with Cx31.1–FLAG and Cx31.1–GFP, no fluorescence recovery was observed, supporting our dual whole-cell patch clamp electrophysiology data indicating that Cx31.1 does not form functional homotypic gap junctions in connexin-deficient keratinocytes (Fig. 8B,C). However, FRAP analysis quantifying the area under the curve revealed that cells expressing Cx31–FLAG and Cx31.1–GFP transferred significantly more Calcein Red-Orange dye when compared to cells with co-expression of Cx31–FLAG and Cx31–GFP or Cx31.1–FLAG and Cx31.1–GFP controls. Consequently, our findings indicate that Cx31–Cx31.1 intermixed gap junctions enhanced GJIC beyond what was achieved by Cx31 expression alone.

## DISCUSSION

Classically, connexins are regarded as gap junction channel-forming proteins that assemble into tightly packed arrays, functioning as conduits for rapid direct intercellular communication. Keratinocytes in the epidermis express up to ten connexin isoforms, which are collectively responsible for regulating epidermal differentiation and renewal through their distinct biophysical properties (Di et al., 2001; Faniku et al., 2015; Goliger and Paul, 1994; Martin et al., 2014; Scott et al., 2012; Martin and van Steensel, 2015; Koval et al., 2014). However, reports on Cx31.1 suggest that this isoform might be GJIC-incompetent when expressed in the absence of other connexins in reference cell types raising questions as to its functional role in tissue-relevant keratinocytes (Hennemann et al., 1992; Bruzzone et al., 1994; Manthey et al., 1999; Manthey et al., 2001; Harris, 2001; Nugent et al., 2021). If Cx31.1 is truly GJIC-incompetent, the question arises whether it can act as a dominant-negative regulator of GJIC or requires assembly with other co-expressed connexin isoforms to enter a functional state. It is notable that Cx31.1 expression has been associated with apoptotic signaling in corneal epithelium, atretic follicles and keratinocytes, suggesting that it regulates this cellular process (Chang et al., 2009; Wright et al., 2001; Nugent et al., 2021). However, the specific canonical and/or non-canonical features of Cx31.1 in the epidermis remain unclear. As such, assessing the life cycle and functional status of Cx31.1 in tissue-relevant keratinocytes might provide insights into the mechanisms by which this connexin isoform uniquely contributes to homeostasis as well as its potential interactions with co-expressed connexin isoforms.

In this study, Cx31.1 was found to occupy intracellular compartments involved in both protein secretion and degradation, accumulate upon proteasome inhibition, and re-localize to perinuclear vesicles upon lysosomal inhibition. Despite being found throughout the secretory pathway, only minimal quantities of Cx31.1 were localized to the cell surface with rare evidence of gap junction-like puncta. Functional studies in GJIC-deficient cells indicate that Cx31.1 fails to form functional homotypic gap junction channels. Interestingly, the relative half-life of Cx31.1 was shorter when Cx43 was present which is possibly a consequence of these connexin isoforms physically interacting where Cx31.1–Cx43 complexes acquired the faster turnover kinetics attributed to Cx43. Alternatively, Cx43-ablated REKs might acquire a cellular phenotype that delays their ability to degrade Cx31.1. Upon co-expression with select keratinocyte connexins, especially Cx31, Cx31.1 could assemble into gap junctions and enhance the levels of GJIC, suggesting that it contributes to functionally active channels.

Unlike Cx43 and other connexins expressed in keratinocytes, we found little evidence that Cx31.1 reached the cell surface. Dual whole-cell patch clamp electrophysiology and FRAP dye transfer assays indicated that Cx31.1 does not form functional homomeric homotypic gap junction channels. These findings in two distinct cell lines are in keeping with other studies where Cx31.1 did not form functional gap junction channels in connexin-deficient *Xenopus* oocytes and HeLa cells (Hennemann et al., 1992; Bruzzone et al., 1994; Manthey et al., 1999, 2001; Harris, 2001; Nugent et al., 2021). Collectively, these findings place Cx31.1 into an unusual category of having little or no ability of being able to form functional gap junction channels when expressed in the absence of other connexins. Although Cx23 is another connexin that fails to form gap junction channels, Cx23 transcripts have not been discovered in primate cDNA libraries suggesting the GJIC incompetency of Cx23 is likely due to gene inactivation, unlike Cx31.1, which has been identified at the protein and transcript level in several human cell types (Sonntag et al., 2009; Berthoud et al., 2016). Thus, Cx31.1 is in a unique category of connexin isoforms exhibiting little capacity to form gap junction channels on its own in tissue-relevant keratinocytes, even though it is present at the mRNA and protein levels in keratinocytes and other cell types (Goliger and Paul, 1994; Di et al., 2001; Kibschull et al., 2004; Shurman et al., 2005; Wiszniewski et al., 2007; Dere et al., 2008; Wang et al., 2009; Serre-Beinier et al., 2009; Zhang et al., 2012; Kibschull et al., 2014; Zhai et al., 2014).

Our Cx31.1 homology models predict that intra- and inter-connexon docking interfaces might form fewer non-covalent bonds compared to what is seen in the experimentally determined Cx26 or Cx32 cryoEM structures. Cx26 and Cx32 are closely related β-subtype connexins that exhibit high sequence identity and similarity to Cx31.1 (Altschul et al., 1997, 2005; Krissinel and Henrick, 2007). The proposed reduction in non-covalent interactions might destabilize the Cx31.1 connexon, hindering oligomerization across sites of cell–cell interactions (Desplantez, 2017; Maeda et al., 2009; Smith et al., 2012). The importance of stabilizing interactions is evident in the V37-V38-A39-A40 structural motif of Cx26, where amino acid substitutions as well as naturally occurring deafness-associated mutations (V37I or A40G) differentially impede gap junction assembly with phenotypic effects ranging from low oligomerization efficiency to non-functional channels (Jara et al., 2012). Interestingly, A39 in this motif is not conserved in Cx31.1, which might contribute to inefficient Cx31.1 oligomerization and poor trafficking to the plasma membrane. Moreover, A39 contributes to the Cx26 hydrophobic core, which stabilizes the protomer (Maeda et al., 2009). Speculatively, the inability of Cx31.1 to form homomeric or homotypic gap junction channels might be partially caused by the low similarity of this structural motif, although, other motifs might also regulate Cx31.1 channel oligomerization and assembly (Martínez et al., 2011; Koval et al., 2014; Smith et al., 2012).

However, the above findings lead to the question of whether Cx31.1 can form intermixed channels with other co-expressed connexin isoforms to overcome the predicted reduction in non-covalent bonds. Cx31.1 homology modeling predicts that its docking interfaces have an ∼20% or ∼15% reduction in the number of hydrogen bonds compared to the number for Cx26 and Cx32, respectively. Thus, it is conceivable that if Cx31.1 co-oligomerized with a compatible connexin isoform like Cx31, apposing hemichannels could theoretically dock, forming heteromeric heterotypic or homotypic channels (Bai et al., 2018). From our studies this appeared to be the case, as keratinocytes co-expressing Cx31.1 and Cx31 had a significantly increased percentage of interfaces with Cx31.1-containing gap junctions and improved GJIC. Other connexin isoforms were less efficient or incapable of this type of rescue, as seen when we co-expressed Cx31.1 with Cx30.3 or Cx43. Nevertheless, to fully understand the structural basis for the limitations on the ability of Cx31.1 to form functional GJ channels, a high-resolution structure of Cx31.1 might be required so as to elucidate key amino acid residues involved in heteromeric oligomerization as well as docking compatibility.

In the event that Cx31.1 does not oligomerize into stable connexons, we postulated that it might accumulate in the ER or Golgi where connexin oligomerization has been shown to occur (Laird, 2006; Koval, 2006). This turned out to not be the case as only low levels of Cx31.1 were found in these compartments. These findings are consistent with the lack of evidence showing that the keratinocytes were under any ER stress and also consistent with a previous report that some Cx31.1–GFP localizes to the ER and Golgi of non-small cell lung cancer cells (Zhang et al., 2012; Zhu et al., 2015). Further assessment of Cx31.1 intracellular distribution revealed that this connexin was likely destined for lysosomal degradation, as suggested in a previous study and as known to be the case for Cx43 (Zhang et al., 2012; Zhu et al., 2015; Laird, 2010). However, the fact that Cx31.1 accumulated in keratinocytes when proteasomes were inhibited suggests that there is crosstalk between degradation pathways that ultimately delays the degradation of Cx31.1.

Given that keratinocytes temporally and spatially express up to ten connexin isoforms in the living layers of the epidermis, we sought to examine whether Cx31.1 would redistribute in response to the co-expression of other keratinocyte connexins or whether Cx31.1 would attenuate and block the trafficking of connexin isoforms that typically form gap junctions. First, we assessed the fate of endogenous Cx43 and co-expressed exogenous Cx43. Here, we found that a population of Cx31.1 appears to interact with endogenous Cx43 within an intracellular compartment, and that this likely contributes to an increased rate of Cx31.1 turnover. Cx43 interacting with β-connexins is not without precedent, as Cx30.3 and mutant Cx26 (H73R or S183F) have been found to bind Cx43 in Cos7 cells and *Xenopus* oocytes, respectively (Okamoto et al., 2020; Shuja et al., 2016). Furthermore, we have recently shown that Cx43 can intermix into the same gap junctions with Cx30.3 and Cx30.3 variants (G12D, T85P and F189Y) in keratinocytes (Lucaciu et al., 2023a). Next, we investigated Cx26, Cx30.3 and Cx31 as potential connexins that could rescue the cell surface delivery of Cx31.1 as they are likely to be expressed within the same keratinocyte and epidermal strata in a healthy epidermis (Aasen and Kelsell, 2009). Notably, Cx31.1 preferentially and abundantly colocalized homogeneously with Cx31 within the same gap junctions providing qualitative evidence that the two isoforms might reside within the same connexon, unlike Cx30–Cx43 and Cx31–Cx43 gap junctions where each connexin occupied a specific subdomain of the gap junction plaque (Kelly et al., 2015; Au et al., 2020). Surprisingly, cells expressing Cx31 and Cx31.1 passed more Calcein Red-Orange dye compared to cells expressing each isoform alone, suggesting these connexins can intermix to elevate GJIC. Interestingly, this observation has been reported previously for heteromeric Cx26 and Cx30 gap junctions which enabled faster intercellular Ca^2+^ signaling compared to their homomeric counterparts (Sun et al., 2005). Collectively, Cx31.1 might crosstalk with co-expressed connexin isoforms in at least three ways. First, a population of Cx31.1 could directly interact with Cx43 and proceed to degrade more rapidly. Second, Cx31.1 putatively interacts with Cx31 (and to a lesser extent Cx26) and might be co-assembled into heteromeric heterotypic gap junction channels. Finally, Cx31.1 could avoid co-expressed connexins like Cx30.3 and exhibit little or no co-assembly. Thus, we speculate that the fate of Cx31.1 is altered through selective connexin interactions, perhaps alluding to its importance in modulating intercellular signaling in a spatial and temporal manner that might be cell differentiation dependent. This raises questions regarding whether these selective interactions with Cx31.1 have pathological implications. Notably, various gain- and/or loss-of-function mutations in five connexins (Cx26, Cx30, Cx30.3, Cx31 and Cx43) have been linked to a variety of congenital skin diseases (Scott et al., 2012; Sellitto et al., 2021; Lucaciu et al., 2023a). As such, it is possible that mutations within these distinct connexin isoforms alter interactions with Cx31.1, which might explain the clinical variability of disease presentation.

In summary, minimal levels of Cx31.1 traffic to the plasma membrane where it fails to assemble into functional gap junction channels when expressed alone in connexin-deficient cells. Based on Cx31.1 homology modeling, we speculate that the GJIC incompetency might reflect insufficient oligomerization, impairing homomeric assembly and thereby trafficking. However, the subcellular localization, relative turnover and gap junction functional status of Cx31.1 was altered through interactions with select co-expressed connexins. Ultimately, this highlights that Cx31.1 might spatially and temporally modulate intercellular signaling in the skin epidermis. As such, it is conceivable that Cx31.1 functions as a differential regulator that has the capacity to dynamically modulate the extent of GJIC in complex cellular systems, such as found in the skin epidermis.

## MATERIALS AND METHODS

### Cell culture

Rat epidermal keratinocytes (REKs) generated from neonatal rat keratinocyte cultures were kindly provided by Vincent C. Hascall (Cleveland Clinic, Cleveland, OH, USA). REKs with a CRISPR/Cas9 ablation of Cx43 were previously described and are referred to here as Cx43 KO REKs (Au et al., 2020). REK and Cx43 KO REKs were incubated in sterile T25 flasks (Fisher Brand, cat. no. FBO12935) at 37°C and 5% CO_2_ in Dulbecco's modified Eagle's medium (DMEM) (Life Technologies, cat. no. 11960-044) (Life Technologies, cat. no. 12430-054) supplemented with 10% fetal bovine serum (Thermo Fisher Scientific, cat. no. 12483-020) and 100 units/ml penicillin-streptomycin (Thermo Fisher Scientific, cat. no. 15140-122). In cases where DMEM did not contain L-glutamine, the growth medium was further supplemented with 2 mM L-glutamine (Thermo Fisher Scientific, cat. no. 25030-081). Cells were seeded into 60 mm tissue culture dishes (Fisher Brand, cat. no. FB012921) containing glass coverslips (Fisher Brand, cat. no. 1254580) for immunofluorescence microscopy studies, 60 mm tissue culture dishes or six-well tissue culture plates (Thermo Fisher Scientific, cat. no. 140675; Fisher Scientific, cat. no. 07-200-83) for immunoblotting, 100 mm tissue culture dishes (Fisher Brand, cat. no. FB012924) for immunoprecipitation and cell surface biotinylation, and 35 mm glass-bottomed dishes (Ibidi, cat. no. 81156) for live-cell imaging.

AD-293 cells (Agilent Technologies, 240085) were used for the dual whole-cell patch clamp functional study of recombinantly expressed gap junctions. To remove all known endogenous connexins (Cx43 and Cx45), CRISPR-Cas9 gene ablation was utilized to nullify human Cx43 in AD-293 cells (Cx43 KO AD-293) as described previously (Esseltine et al., 2020). Human Cx45 was subsequently deleted by transiently transfecting Cx43 KO AD-293 cells with the pSpCas9(BB)-2A-GFP plasmid (Addgene, cat. no. #48138) using the X-tremeGENE HP DNA transfection reagent (Roche Diagnostics GmbH, cat. no. 06366546001) as described previously (Wong et al., 2024). On the second day after transfection, cells were maintained under puromycin (MilliporeSigma, cat. no. P8833) selection pressure for 3 days; 5 μg/ml puromycin was added to DMEM (Life Technologies, cat. no. 10313-021) supplemented with 10% fetal bovine serum (Life Technologies, cat. no. 080150), 1% penicillin-streptomycin (Life Technologies, cat. no. 15140-122) and 1% GlutaMAX (Life Technologies, cat. no. 35050-061) in an incubator with 5% CO_2_ at 37°C. Double knockout Cx43 and Cx45 AD-293 cells were routinely assessed for junctional coupling between adjacent cells as a negative control, and functional coupling was never observed throughout our experiments.

### Plasmids

The constructs encoding human Cx31.1 and Cx31 [pCMV6-Cx31.1- Myc-DDK (cat. no. RC202521) and pCMV6-Cx31-Myc-DDK (cat. no. RC204405)] were purchased from OriGene Technologies, Inc. Engineering of Cx31.1–GFP and -pIRES-GFP vectors was outsourced to NorClone Biotech Laboratories (London, Ontario, Canada) and generated from a restriction digest of pCMV6-Cx31.1-Myc-DDK to isolate Cx31.1 cDNA which was subcloned into pEGFP N and pIRES2-GFP vectors, respectively. The generation of GFP-tagged Cx30.3, Cx26, Cx30 and Cx43 was described previously (Berger et al., 2014; Beach et al., 2020). The cDNA of mouse Cx50 was a generous gift from Dr David Spray (Albert Einstein College of Medicine, Bronx, NY, USA) as described previously (Xin et al., 2010). Cx50-IRES-GFP generation was described in our earlier paper (Tong et al., 2014).

### Transfection

REKs and Cx43 KO REKs were seeded and grown to ∼60–80% confluence and transiently transfected with cDNA using Lipofectamine 3000 (Thermo Fisher Scientific, cat. no. L3000015). For each transfection the complexation components were combined as described previously (Au et al., 2020). Experiments were conducted at 24–48 h following transient transfection. In cases where Cx43 KO REKs were transfected with combinations of Cx26–GFP, Cx30.3–GFP, Cx31–GFP, Cx31–FLAG, Cx31.1–GFP, Cx31.1–FLAG or Cx43–GFP, equal DNA concentrations of each plasmid were combined.

AD-293 cells were transfected with 0.6 μg of cDNA, and 1.2 μl of X-tremeGENE HP DNA transfection reagent (Roche Diagnostics GmbH, cat. no. 06366546001) at a 1:2 ratio of cDNA to transfection reagent in Opti-MEM medium (Life Technologies, cat. no. 51985-034) for 5 h. The transfected cDNA included either a co-transfection of 0.5 μg of Cx50 or Cx31.1–FLAG, and 0.1 μg of IRES-EGFP at a 5:1 construct to reporter ratio, or 0.6 μg of Cx50-IRES-EGFP, Cx31.1-IRES-EGFP or IRES-EGFP. After transfection, the medium was then replaced with FBS-containing DMEM, and cells were incubated overnight. Transfected AD-293 cells were replated onto glass coverslips the following day and left for at least 3 h before transfer to a recording chamber for dual whole-cell patch clamp electrophysiology. Cell pairs successfully transfected with IRES-EGFP (in the case of co-transfection), or Cx50-IRES-EGFP or Cx31.1-IRES-EGFP were selected for the functional study of homotypic gap junction channels.

### Immunolabeling and imaging

REKs and Cx43 KO REKs grown on 12 mm glass coverslips were fixed using an ice-cold solution of 80% methanol and 20% acetone for 10 min at 4°C, or with 4% paraformaldehyde (PFA) for 15 min at room temperature. Cells were washed three times with 1× phosphate-buffered saline (PBS) (Fisher Brand, cat. no. 14190250) and if necessary stored submerged in 1× PBS at 4°C. PFA-fixed cells were permeabilized with 0.25% Triton X-100 in 1× PBS for 10 min at room temperature. Cells were blocked with 2% bovine serum albumin (BSA; BioShop, ALB001) in 1× PBS for 30 min at room temperature in a humidity chamber. Cells were incubated for 1 h at room temperature with various primary antibodies: mouse anti-FLAG (2 g/ml; 1:500; MilliporeSigma, cat. no. F3165), mouse anti-PDI (5 g/ml; 1:200; Enzo Life Science, cat. no. ADI-SPA-891F), mouse anti-GM130 (1.25 g/ml; 1:200; BD Transduction Laboratories, cat. no. 610822), mouse anti-MTCO1 (5 g/ml; 1:200; Abcam, cat. no. 1D6E1A8), rabbit anti-FLAG (0.7 g/ml; 1:200; Cell Signaling, cat. no. 14793), rabbit anti-Cx43 (1–1.6 g/ml; 1:500; MilliporeSigma, cat. no. C6219), rabbit anti- LAMP-2A (2.5 g/ml; 1:100; Thermo Fisher Scientific, cat. no. 51-2200), rabbit anti-Rab7 (0.02 g/ml; 1:100; Cell Signaling, cat. no. 9367 T) or rabbit anti-LC3B (10 g/ml; 1:100; Thermo Fisher Scientific, cat. no. PA1-46286). Cells were subsequently washed three times with 1× PBS and incubated at room temperature for 1 h with secondary antibodies: goat anti-mouse IgG Alexa Fluor™ 555 (2.5 g/ml; 1:800; Invitrogen, cat. no. A21422), goat anti-rabbit IgG Alexa Fluor™ Plus 555 (2.5 g/ml; 1:800; Invitrogen, cat. no. A32732), donkey anti-mouse IgG Alexa Fluor™ 488 (2.5 g/ml; 1:800; Invitrogen, cat. no. A21202) or donkey anti-rabbit IgG Alexa Fluor™ 488 (2.5 g/ml; 1:800; Invitrogen, cat. no. A21206). In some cases, cells were labeled with Alexa Fluor™ 633-conjugated wheat germ agglutinin (WGA) (1.25 mg/ml; 1:100; Thermo Fisher Scientific, cat. no. W21404). Labeled cells were washed three times with 1× PBS and incubated with Hoechst 33342 (10 g/ml; 1:1000; Thermo Fisher Scientific, cat. no. H3570) for 5 min. Coverslips were mounted onto microscope slides (Fisher Brand, cat. no. 22034486) using Airvol mounting medium. Confocal images were captured on a Zeiss LSM 800 Airyscan confocal microscope using a plan-apochromat (63×, 1.4 NA) oil lens. The line intensity profile of fluorescence images was quantified using Fiji ImageJ software (Schindelin et al., 2012). Lines were randomly selected for quantification. When quantifying gap junctions, two third-party investigators who were not aware of the experimental condition quantified the percentage of cell interfaces with Cx31.1-containing gap junction plaques, which was averaged for subsequent statistical assessment. Gap junction plaques were defined as linear or punctate 0.2 μm or larger regions of red or green fluorescence signal at the interface of two transfected cells.

### Live-cell imaging

Cx43 KO REKs expressing Cx31.1–GFP were grown on 35 mm glass-bottomed dishes (Ibidi, cat. no. 81156). Prior to imaging, cells were incubated with Hoechst 33342 (1:2000 in 1× PBS) for 5 min in an incubator with 5% CO_2_ at 37°C. Cells were subsequently washed with 1× PBS and bathed in Phenol Red-free DMEM (Thermo Fisher Scientific, cat. no. 31053-028) supplemented with 10% fetal bovine serum (Thermo Fisher Scientific, cat. no. 12483-020), 100 units/ml penicillin-streptomycin (Thermo Fisher Scientific, cat. no. 15140-122) and 2 mM L-glutamine (Thermo Fisher Scientific, cat. no. 25030-081). The dynamics of Cx31.1 were visualized using a Zeiss LSM 800 Airyscan confocal microscope. Images were acquired every 25–35 s for up to 30 min at 37°C and 5% CO_2_ as previously described (Kelly et al., 2015).

### Fluorescence recovery after photobleaching

Non-transfected Cx43 KO REKs or cells expressing a combination of Cx31–GFP, Cx31–FLAG, Cx31.1–GFP and Cx31.1–FLAG grown on 35 mm glass-bottomed dishes were loaded with 7.5 μg/ml CellTrace™ Calcein Red-Orange, AM (Thermo Fisher Scientific, cat. no. C34851) in Opti-MEM Reduced Serum Medium (Thermo Fisher Scientific, cat. no. 31985-070) for 30 min at 37°C and 5% CO_2_. Dye-loaded cells were washed three times with 1× PBS and bathed in Opti-MEM. Using a Zeiss LSM 800 Airyscan confocal microscope, an objective lens heating collar was used in conjunction with a CTi controller 3700 digital system to maintain physiological temperatures of 37°C while bathing the cells in humidified air and 5% CO_2_. Randomly selected GFP-positive cells were photobleached with a 561-nm argon laser to ∼30% residual fluorescence. Calcein Red-Orange recovery into the photobleached cells was tracked by repeated 1 s image acquisition and quantified using ZEISS ZEN 2.3 software.

### Immunoblotting

REKs and Cx43 KO REKs were lysed on ice with 1× RIPA lysis buffer (50 mM Tris-HCl,150 mM NaCl, 1% NP-40, 0.5% sodium deoxycholate and 0.1% SDS) supplemented with 100 mM NaF (BDH, cat. no. BDH9290-500G), 100 mM Na_3_VO_4_ (Thermo Fisher Scientific, cat. no. 13721-39-6), and a cOmplete™ mini protease inhibitor cocktail tablet (MilliporeSigma, cat. no. 11836153001) as described previously (Lucaciu et al., 2022). Cell lysates were subsequently centrifuged at 10,000 ***g*** at 4°C for 15 min and supernatants were collected for bicinchoninic acid (BCA) assay (Thermo Fisher Scientific, cat. no. 23277). Proteins were resolved by SDS-PAGE and transferred to nitrocellulose membranes and blocked with 3% BSA in PBS with 0.05% Tween-20 (PBST) for 30 min at room temperature. Blots were incubated overnight at 4°C with various primary antibodies: rabbit anti-Cx43 (0.1–0.16 g/ml; 1:5000; MilliporeSigma, cat. no. C6219), rabbit anti-FLAG (0.047 g/ml; 1:3000; Cell Signaling, cat. no. 14793), rabbit anti-GAPDH (0.1 g/ml; 1:1000; MilliporeSigma, cat. no. G9545), rabbit anti-binding immunoglobulin protein (BiP)/GRP78 (5–7.5 g/ml; 1:2000; MilliporeSigma, cat. no. G8918), mouse anti-FLAG (0.02 g/ml; 1:5000; MilliporeSigma, cat. no. F3165) or mouse anti-GAPDH (0.2 g/ml; 1:5000; MilliporeSigma, cat. no. MAB374). The following day, nitrocellulose membranes were washed three times with PBST and incubated for 45 min at room temperature with secondary antibodies: goat anti-mouse-IgG IRDye^®^ 800CW (0.05 g; 1:10,000; LICOR, cat. no. 926-32210), goat anti-mouse-IgG IRDye^®^ 680RD (0.05 g; 1:10000; LICOR, cat. no. 926-68070), goat anti-rabbit-IgG IRDye^®^ 800CW (0.05 g; 1:10,000; LICOR, cat. no. 926-32211) and goat anti-rabbit-IgG IRDye^®^ 680RD (0.05 g; 1:10,000; LICOR, cat. no. 926-68071). An Odyssey LiCor infrared imaging system was utilized to detect proteins of interest and the BLUelf Prestained Protein Ladder molecular mass markers (FroggaBio, cat. no. PM008). Quantification was performed using the Odyssey LiCor infrared imaging system and in cases where the levels of Cx31.1-FLAG were assessed only the monomer species were considered for quantification. Full presentations of immunoblots are shown in Fig. S8.

### Cell viability assay

A colorimetric MTT assay quantifying the reduction of MTT [3-(4,5-dimethylthiazol-2-yl)-2,5-diphenyltetrazolium bromide] to formazan in REKs and Cx43 KO REKs was utilized to assess how Cx31.1–FLAG influences cell viability 24 h following the transfections. MTT (Abcam, cat. no. ab211091) was added to the culture medium to a final concentration of 2.5% (v/v) and incubated at 37°C and 5% CO_2_ for 1 h. Following incubation, the growth medium mixture was aspirated, and the insoluble formazan product produced was dissolved in 1 ml DMSO (Thermo Fisher Scientific, cat. no. J66650-AK) at room temperature for 5 min with gentle shaking. The optical density at 570 nm (OD570) was assessed using a Victor3 multiplate reader. As a control, non-transfected cells were treated with or without 1 mM staurosporine (Sigma, cat. no. S6942).

### Cell treatments

To assess the consequences of inhibiting proteasomal or lysosomal degradation on the fate of connexins, at 24 h following the expression of Cx31.1, cells were treated for 4 or 10 h with the proteasomal inhibitor MG132 (50 µM; Cell Signaling, cat. no. 2194S) or the lysosomal inhibitors ammonium chloride (10 mM; Thermo Fisher Scientific, cat. no. A661-500) or chloroquine (100 µM; MilliporeSigma, cat. no. C6628). To assess the relative turnover of Cx31.1 and/or Cx43, at 24 h following the expression of Cx31.1, cells were treated with cycloheximide (10 µg/ml; MilliporeSigma, cat. no. 239764) for 0, 1, 3, 5 or 7 h. Drug-treated cells were lysed and subjected to immunoblotting as described above.

### Cell surface biotinylation

REKs transiently expressing Cx31.1–FLAG in 100 mm dishes were subject to cell surface biotinylation at 24 h following transfection. All experimental proceedings were completed on ice or at 4°C. Cell monolayers were washed with three times with cold 1× PBS that was supplemented with 100 mM NaF and 100 mM Na_3_VO_4_. Control and Cx31.1-expressing cells were subsequently incubated on a shaker with 1× PBS with or without 0.4 mg/mL EZ-Link™ Sulfo-NHS-SS-Biotin (Thermo Fisher Scientific, cat. no. A39258) for 20 min at 4°C. In order to quench reactive biotin, cell monolayers were rinsed twice with 1× PBS containing 100 mM glycine (BioShop, cat. no. 56-40-6) and incubated on a shaker with the same reagent for 30 min at 4°C. Cells were disrupted in SDS lysis buffer (1% Triton X-100, 0.1% SDS, 100 mM NaF, 100 mM Na_3_VO_4_ in PBS) on a shaker for 1 h at 4°C. Lysed cells were scraped from the tissue culture dishes and rocked for an additional 30 min at 4°C. Cell lysates were subsequently centrifuged at 10,000 ***g*** at 4°C for 15 min and supernatants were collected for BCA-based assessment of protein content. For the cell surface biotinylation pulldown, 750 μg of protein lysates were combined with 50 μl of Pierce™ streptavidin magnetic beads (Thermo Fisher Scientific, 88817). Magnetic beads were pre-washed with 1× IP buffer (10 mM Tris-HCl pH 7.4, 150 mM NaCl, 1 mM EDTA, 1 mM EGTA, 0.5% NP-40 and 1% Triton X-100) supplemented with 100 mM NaF and 100 mM Na_3_VO_4_ and resuspended in the same mixture. Input and pulldown lysates were rocked overnight at 4°C. The beads were subsequently washed three times with 1× IP buffer and resuspended in 2× Laemmli sample buffer (BioRad, cat. no. 1610747). Resuspended beads in parallel with 25 μg of input protein lysates in 2× Laemmli sample buffer were boiled for 5 min. Proteins were then resolved by SDS-PAGE as described earlier. Immunoblotting for E-cadherin (0.1 μg/ml; 1:2500; BD Transduction Laboratories™, cat. no. 610182) and GAPDH were used as positive and negative controls, respectively.

### Immunoprecipitation

REKs transiently expressing Cx31.1–FLAG were subject to immunoprecipitation 24 h following transfection. Cells were lysed on ice with 1× IP buffer supplemented with 100 mM NaF, 100 mM Na_3_VO_4_, and a cOmplete™ mini protease inhibitor cocktail tablet. Cell lysates were collected for a BCA protein assessment. 25 µl (0.75 mg) of Pierce™ Protein G Magnetic Beads (Thermo Fisher Scientific, cat. no. 88847) were collected and the supernatant was isolated using a DynaMag-2. The supernatant was discarded, and beads were resuspended and subsequently rotated with the corresponding antibodies for immunoprecipitation for 10 min at room temperature; rabbit anti-FLAG (1.41 μg/ml; 1:100; Cell Signaling, cat. no. 14793) or rabbit anti-Cx43 (2.5–4 μg/ml; 1:200; MilliporeSigma, cat. no. C6219). Antibodies were diluted in 200 µl of PBST. As a control, beads were resuspended with 200 µl of PBST or in some cases rabbit anti-IgG (10 μg/ml; 1:500; Thermo Fisher Scientific, cat. no. 02-6102). Following this, the beads were washed three times with PBST and resuspended with 1000 μg of protein lysate and rotated at room temperature for 30 min. Unbound proteins were discarded, and the beads were washed three times with PBST. The beads were then resuspended in 20 µl of glycine (50 mM, pH 2.8) and 10 µl 2× Laemmli sample buffer to elute the target antigen. All samples were then heat to 70°C for 10 min using a BioRad T100 thermal cycler. Proteins were then resolved by SDS-PAGE as described earlier.

### Dual whole-cell patch clamp

Glass coverslips with transfected AD-293 cells were placed into a recording chamber on an upright microscope (BX51WI, Evident Corporation, Shinjuku, Tokyo, Japan). The chamber was filled with extracellular solution (ECS), containing (in mM): 135 NaCl, 2 CsCl, 2 CaCl_2_,1 MgCl2, 1 BaCl_2_, 10 HEPES, 5 KCl, 5 D-(+)-glucose, 2 sodium pyruvate, pH adjusted to 7.4 with 1 M NaOH, and osmolarity of 310–320 mOsm. Isolated cell pairs expressing GFP (in the case of co-transfection), or Cx50-IRES-EGFP or Cx31.1-IRES-EGFP were selected for dual whole-cell patch clamp. Dual whole-cell patch clamp was performed at room temperature (22–24°C) using a MultiClamp 700A amplifier (Molecular Devices, Sunnyvale, CA, USA) as previously described (Wong et al., 2024). A micropipette puller (PC-100, Narishige International, Amityville, NY, USA) was used to pull patch pipettes, which were filled with intracellular solution (ICS) containing (in mM): 130 CsCl, 10 EGTA, 0.5 CaCl_2_, 3 MgATP, 2 Na_2_ATP, 10 HEPES, adjusted to pH 7.2 with 1 M CsOH, and osmolarity of 290–300 mOsm. Each cell in a selected pair was initially voltage clamped at 0 mV. Voltage pulses were applied to one cell of the pair to establish the transjunctional voltage (*V*_j_), while the other cell of the pair was constantly held at 0 mV to record gap junctional current (*I*_j_). The current was low-pass filtered (Bessel filter at 1 kHz) and recorded using Clampex10.7 software at a sampling frequency of 10 kHz via an AD/DA converter (Digidata 1550, Molecular Devices, Sunnyvale, CA, USA).

### Generation and assessment of Cx31.1 homology models

Human Cx31.1 (primary accession number O95377) dodecamer models were generated in MODELLER (version 10.4) using the cryoEM-determined human Cx32 structure (PDB ID: 7ZXM) or human Cx26 structure (PDB ID: 7QER) as the templates (Brotherton et al., 2022; Webb and Sali, 2021; Qi et al., 2023). The sequences were aligned with Jalview 2.11.2.6 using ClustalO with defaults. The Cx32 structure was selected as the template given that it has been resolved to a high-resolution (i.e. 2.14 Å; 1 Å= 0.1 nm) and has a comparable sequence to Cx31.1 (53% and 73% identity and similarity, respectively) (Qi et al., 2023). Similarly, the Cx26 structure was solved to a high-resolution (i.e. 2.20 Å) resolving a further 11 more residues on the N-terminal side of the protein and has a comparable sequence to Cx31.1 (49% and 69% identity and similarity, respectively) (Brotherton et al., 2022). As the Cx26 7QER structure resolves more amino acid residues, this structure was utilized as a template to model Cx31.1–Cx31 and Cx31.1–Cx26 intermixed channels. For each of the gap junction channels, ten homology models were generated with no symmetry restraints enforced. The models with the lowest discrete optimized protein energy (DOPE) score were taken for visualization and structural analyses with the Protein Interfaces, Surfaces and Assemblies (PISA) tool (Krissinel and Henrick, 2007). The diameter of the aqueous pore of gap junction channel models was estimated at 3 Å steps along the length of the channel using PoreWalker 1.0 and the pore bottleneck was identified via the narrowest estimated pore diameter (Pellegrini-Calace et al., 2009). En face and cross-section views of gap junction channel models were generated using Chimera X v1.6.1 (Meng et al., 2023; Pettersen et al., 2021). The pdb coordinate files of all homology models are available from figshare.com (see Data Availability section for more details).

### Sequence analysis

Protein sequences for human Cx26 (primary accession number P29033), Cx30.3 (primary accession number Q9NTQ9), Cx31 (primary accession number O75712), Cx32 (primary accession number P08034) and Cx43 (primary accession number P17302) were analyzed using BlastP pairwise sequence alignment relative to the Cx31.1 (primary accession number O95377) query sequence (Altschul et al., 1997, 2005). The amino acid identity and similarity were recorded while accounting for sequence gap size variability due to differences in connexin protein length.

### Statistical analysis

Data is depicted as mean±s.e.m. and analyzed using a two-tailed unpaired Student's *t*-test or Mann–Whitney test for analysis between two groups and a one-way ANOVA test with Dunnett's or Tukey post test. The percentage of cell pairs coupled, and junctional conductance data obtained from dual whole-cell patch clamp electrophysiology are depicted as the median with the error bars representing the interquartile range and analyzed between groups using a Mann–Whitney test. GraphPad Prism Version 8 (GraphPad Software, San Diego, California, www.graphpad.com) was utilized for the completion of all statistical analysis.

## Supplementary Material

10.1242/joces.261631_sup1 Supplementary information
