# Supplementary material for: Cx31.1 can selectively intermix with co-expressed connexins to facilitate its assembly into gap junctions
Source: J Cell Sci. 2024 Apr 17;137(7):jcs261631. doi: 10.1242/jcs.261631 (PMC11058089; doi:10.1242/jcs.261631)
Supplement: Supplementary information [file joces-137-261631-s1.pdf]

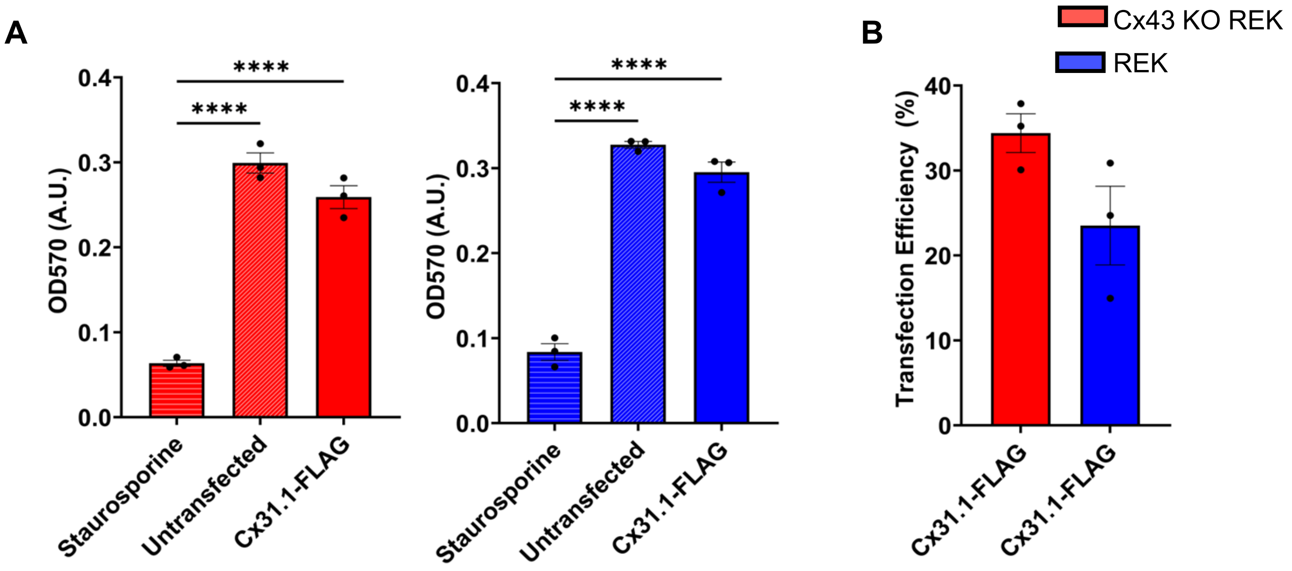

Fig. S1. Cx31.1 does not alter keratinocyte cell viability. (A) A MTT assay indicated that Cx31.1-FLAG expressing cells were not prone to cell death. As a control, untransfected REKs were treated with 1 mM staurosporine. N=3, one-way ANOVA; ****P < 0.0001. (B) Quantification of Cx31.1-FLAG transfection efficiency of REKs and REKs lacking Cx43 completed in parallel indicated that both populations of keratinocytes expressed Cx31.1 at a statistically similar rate. N=3, student's t-tests; not significant.

Journal of Cell Science • Supplementary information

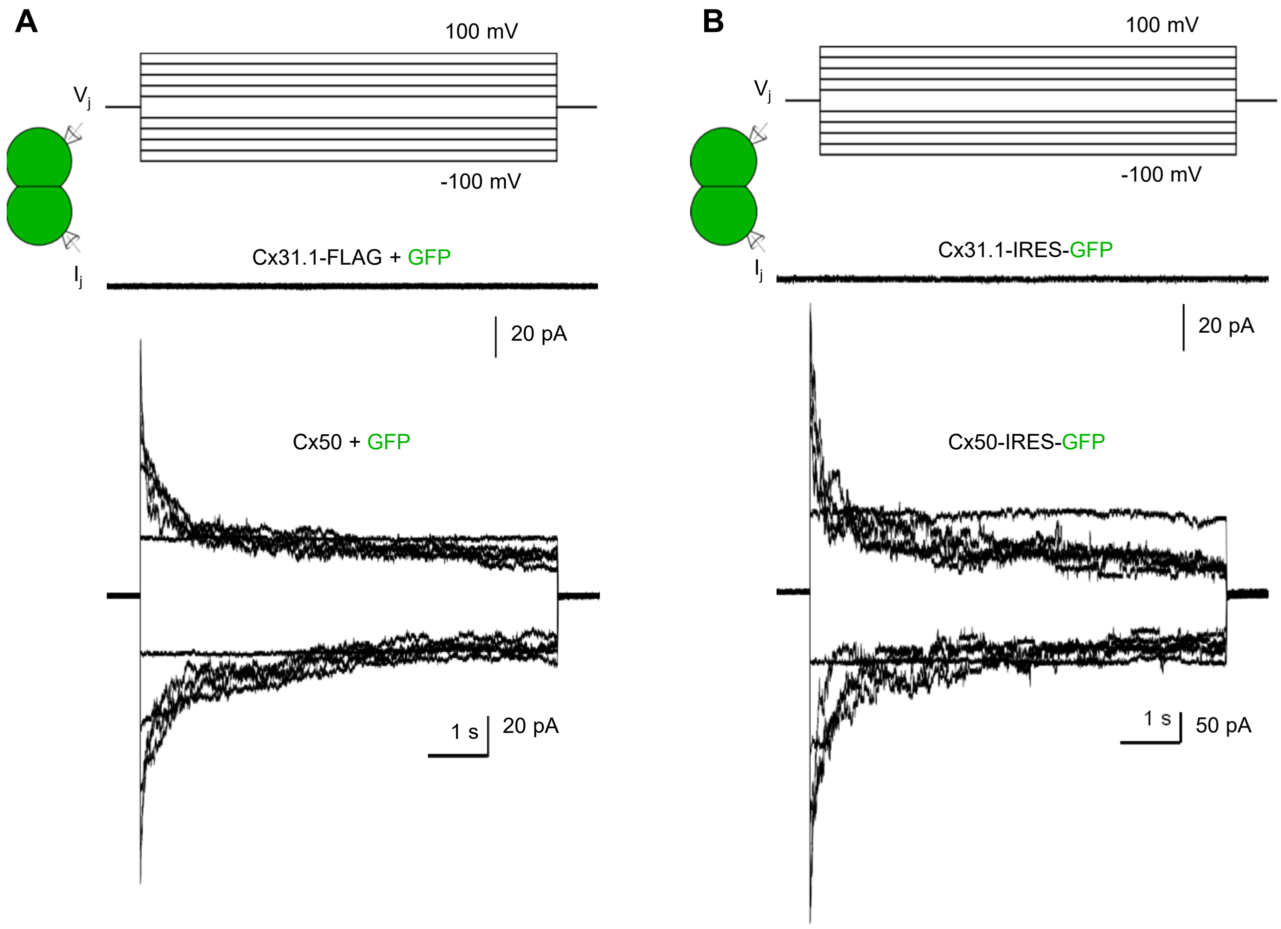

Fig. S2. Macroscopic junctional analysis revealed that Cx31.1 is unable to form voltage-gated channels. Superimposed junctional currents (Ij) measured in response to a series of transjunctional voltage (Vj) pulses (±20 to ±100mV with 20mV increments) recorded in AD-293 cell pairs lacking Cx43 and Cx45 but projected to co - express (A) GFP in combination with Cx31.1-FLAG or Cx50 (control) (N=4) or express (B) Cx31.1-IRES-GFP or Cx50-IRES-GFP (control) (N=3). Macroscopic currents indicate that Cx31.1 fails to demonstrate voltage gating unlike what is seen in functional Cx50 homotypic gap junction channels.

Journal of Cell Science • Supplementary information

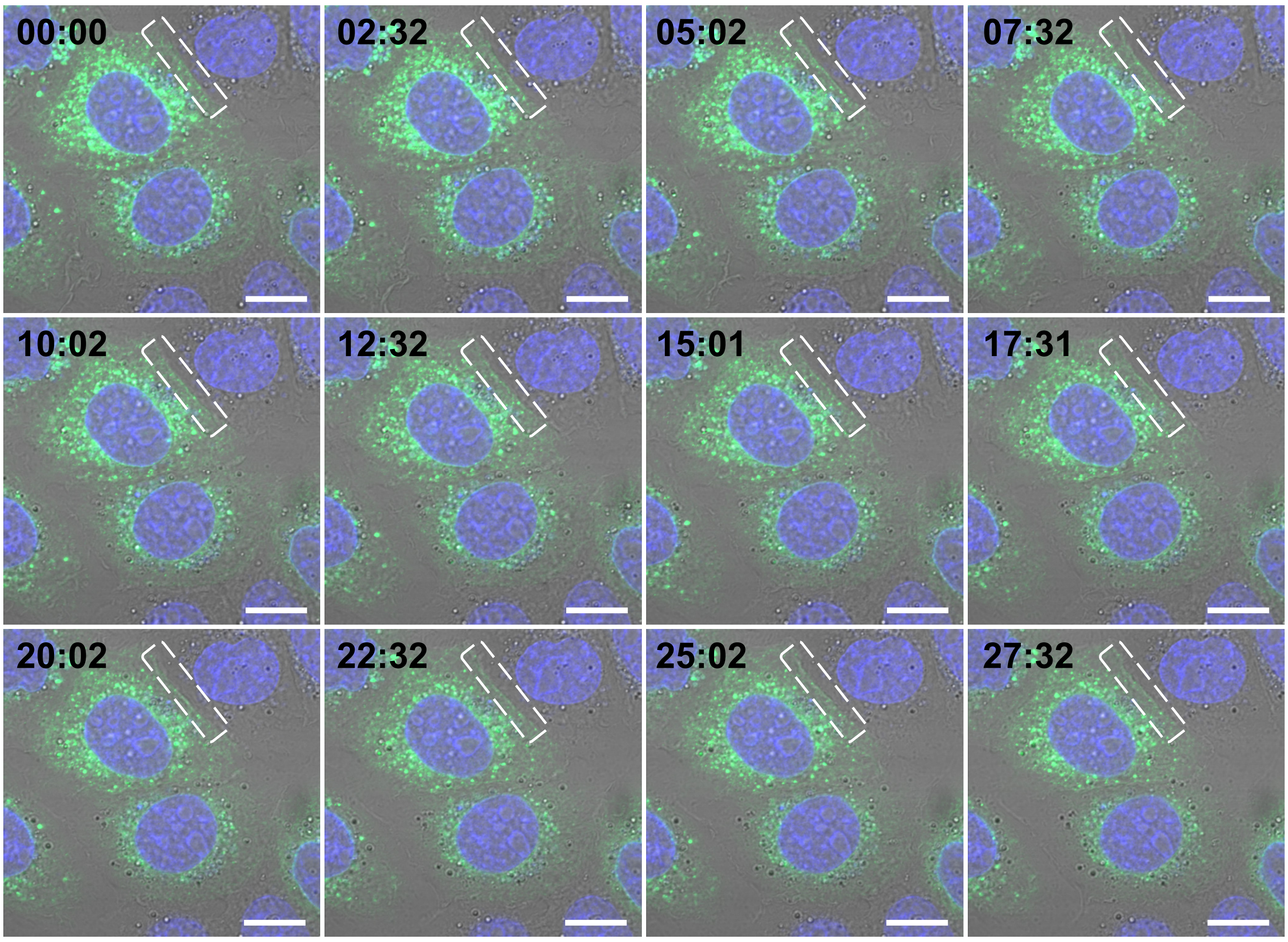

Fig. S3. Live-cell imaging reveals a subpopulation of cells where Cx31.1-GFP can be seen at the cell surface. Live-cell time-lapse images of Cx43 KO REKs expressing Cx31.1-GFP (green). Images of Cx31.1-GFP were acquired at ~ 3 0 second intervals for nearly 28 minutes. Cx31.1-GFP was identified at the plasma membrane of a few Cx31.1-GFP expressing cells where it remained for the duration of the recording period (dashed box). The plasma membrane was identified via differential interface contrast imaging. Nuclei were stained with Hoechst 33342 (blue). Scale bars = 10 µm. N=3.

Journal of Cell Science • Supplementary information

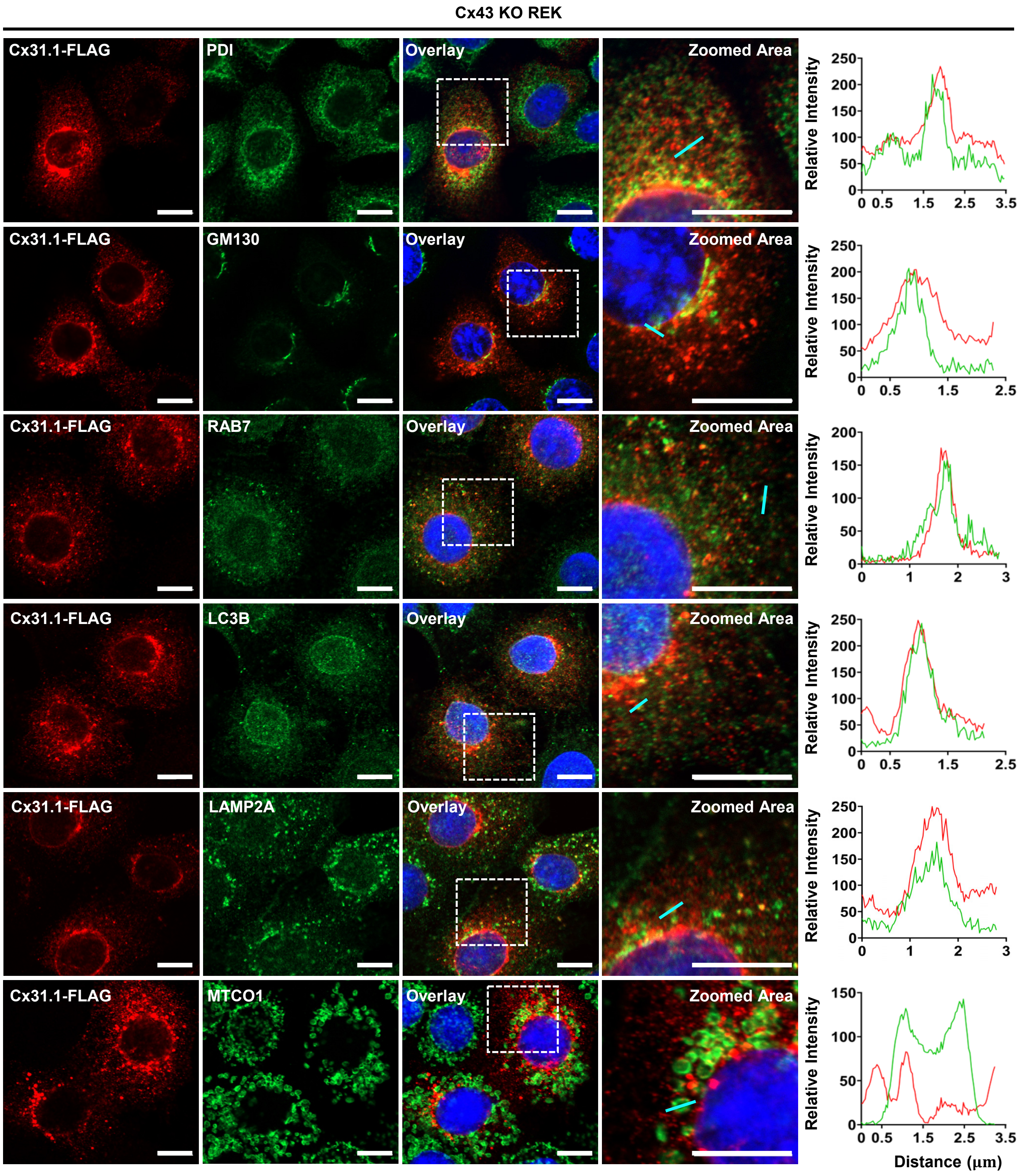

Journal of Cell Science • Supplementary information

Fig. S4. Cx31.1 localizes to multiple intracellular compartments in Cx43 KO REKs. Cx43 KO REKs expressing Cx31.1-FLAG (red) partially co-localize with resident proteins of the ER (green: α-PDI), Golgi apparatus (green: α-GM130), late endosomes (green: α-Rab7), autophagosomes (green: α-LC3B), and lysosomes (green: α-LAMP-2A), suggesting Cx31.1-FLAG enters intracellular compartment associate with protein secretion and degradation. Cx31.1 failed to co-localize with mitochondria as denoted by the mitochondrial marker (green: α-MTCO1). Dashed boxes denote zoomed imaged areas. The fluorescent intensity profile of each representative image was quantified along the cyan line. Nuclei are stained with Hoechst 33342 (blue). Scale bars = 10 μm. N=3.

Journal of Cell Science • Supplementary information

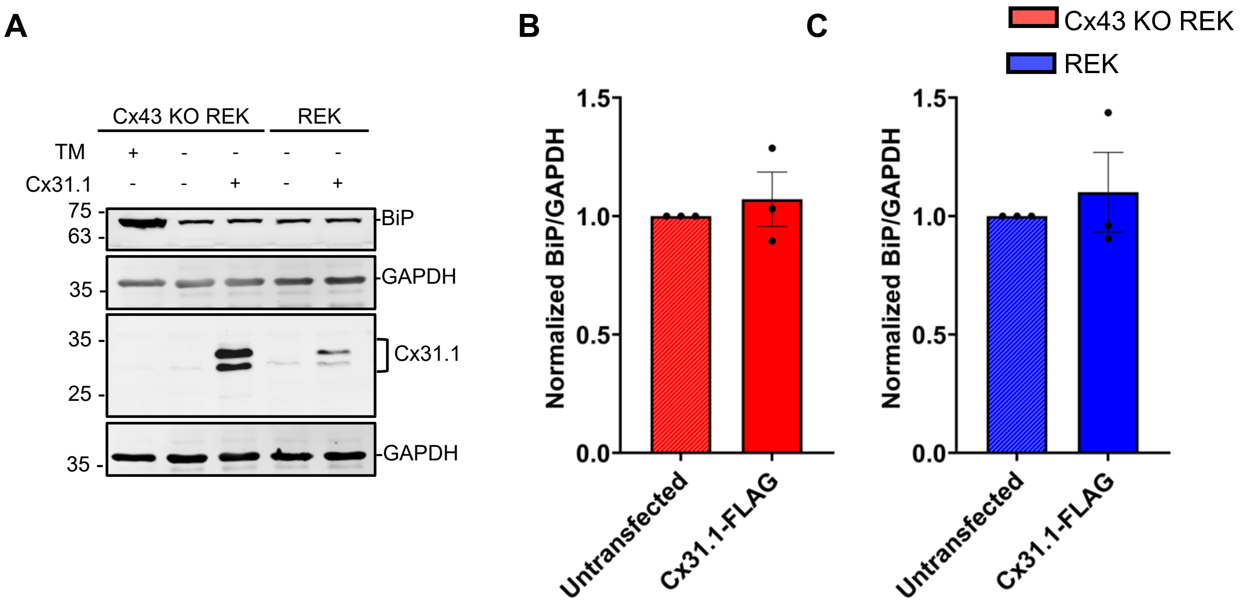

Fig. S5. Intracellular Cx31.1 does not elevate the ER stress indicator BiP. (A) Representative immunoblots of Cx31.1-FLAG expressing REKs and Cx43 KO REKs probed for binding immunoglobulin protein (BiP). As a positive control Cx43 KO REKs were treated with 2 μg/mL tunicamycin (TM). (B) Quantification of BiP levels in Cx43 KO REKs and (C) REKs revealed that Cx31.1 expression does not elevate the ER stress indicator BiP. N=3, protein size in kDa, student's t-tests; not significant.

Journal of Cell Science • Supplementary information

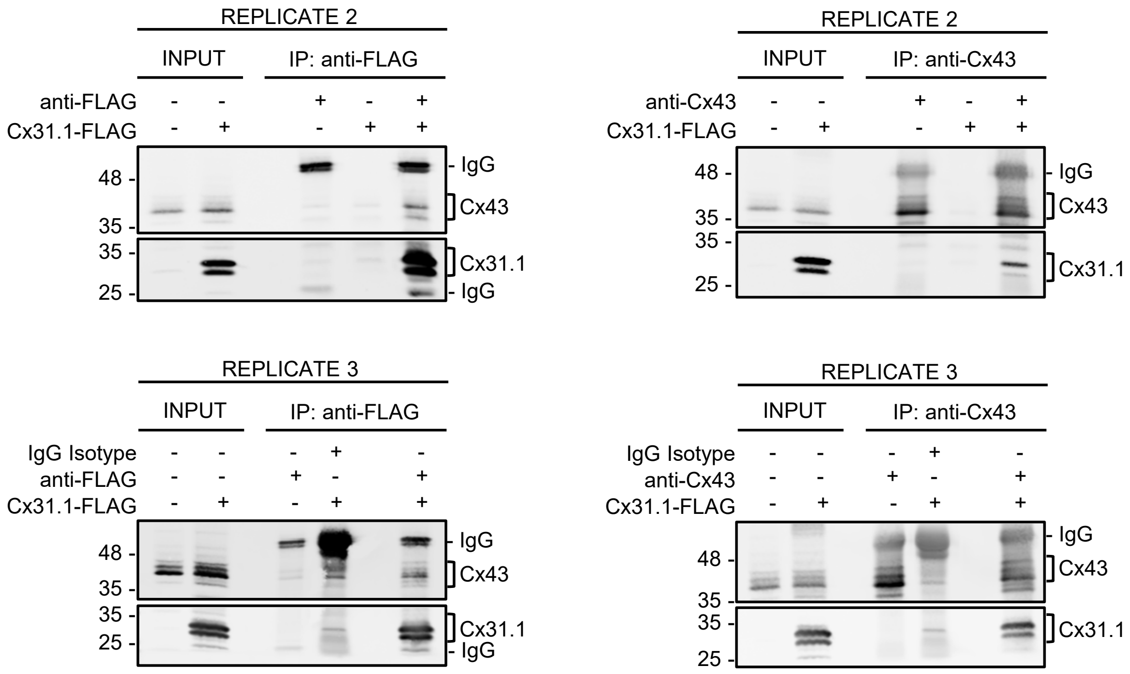

Fig. S6. Cx31.1-FLAG interacts with endogenous Cx43. Replicate two and three of REKs expressing Cx31.1-FLAG were immunoprecipitated (IP) with α-FLAG or α-Cx43. To control for non-specific pulldown of the target protein, lysates were assessed in the absence of α-FLAG or α-Cx43 antibodies with either an antibody null beads only control (0.05% PBS-T) or isotype control (α-IgG). Endogenous Cx43 was co-immunoprecipitated with α-FLAG and Cx31.1-FLAG with α-Cx43 suggesting these isoforms interact. Note low levels of non-specific binding of Cx31.1 and/or Cx43 to the protein G beads used in these studies. N=3.

Journal of Cell Science • Supplementary information

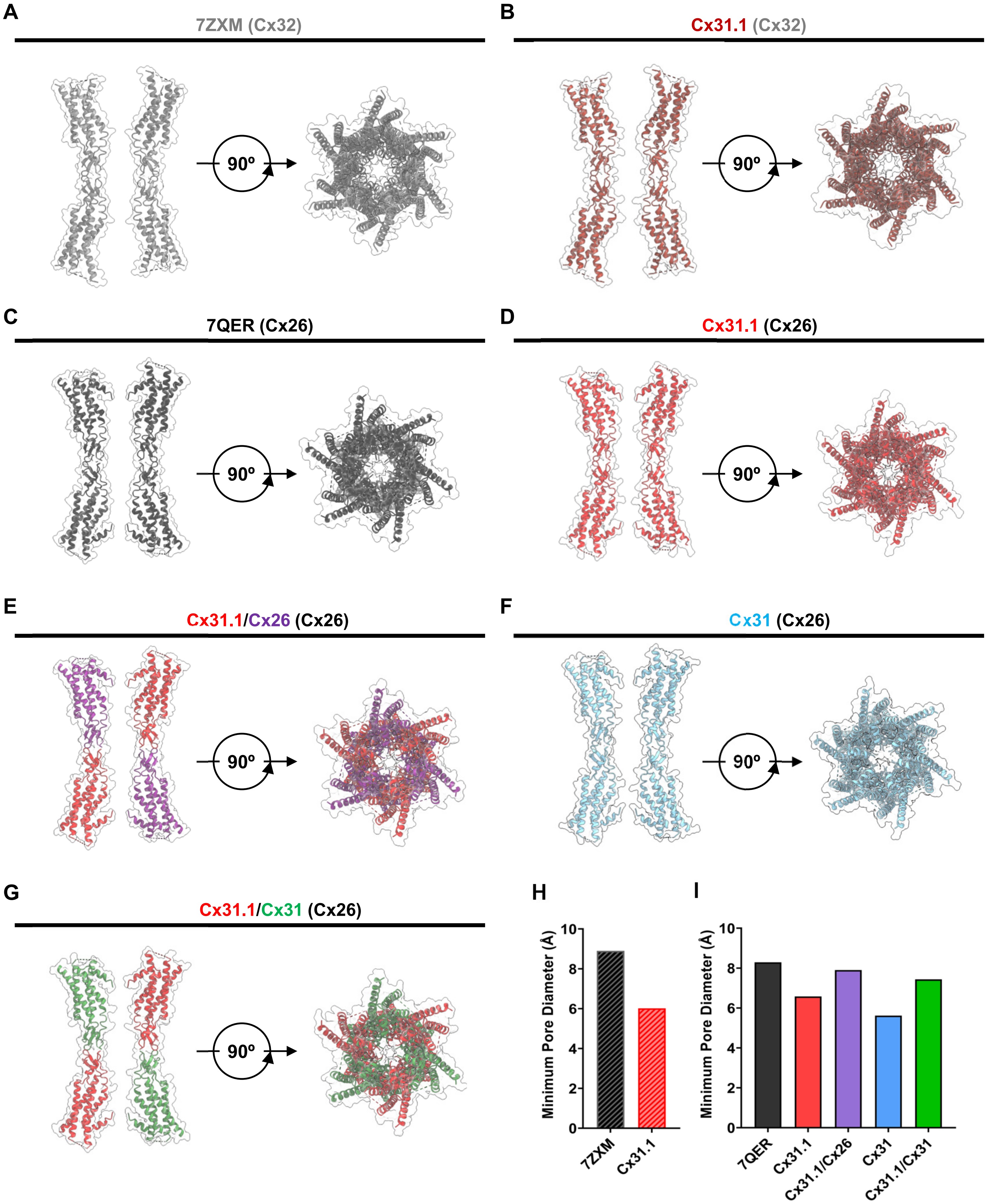

Journal of Cell Science • Supplementary information

Fig. S7. Cx31.1 homotypic homology models are predicted to have a smaller minimum pore diameter when compared to the corresponding template structure and heterotypic Cx31.1 intermixed homology models. *En face* and cross-section views of (A) Cx32 (PDB ID: 7ZXM) and the corresponding (B) Cx31.1 homology model dodecamer. (H) PoreWalker 1.0 predicts that the Cx31.1 homology model has a smaller minimum pore diameter when compared to the template structure 7ZXM. *En face* and cross-section views of (C) Cx26 (PDB ID: 7QER) and the corresponding homology model dodecamer models of (D) homomeric/homotypic Cx31.1 (E) heteromeric/heterotypic Cx31.1/Cx26 (F) homomeric/homotypic Cx31 and (G) heteromeric/heterotypic Cx31.1/Cx31. (I) PoreWalker 1.0 predicts that the Cx31.1 homomeric/homotypic homology model has a smaller minimum pore diameter when compared to the template structure 7QER, heteromeric/heterotypic Cx31.1/Cx26, and heteromeric/heterotypic Cx31.1/Cx31. All structures were generated using Chimera X Version 1.6.1.

Fig. 2 Full Blots

*= Displayed in manuscript

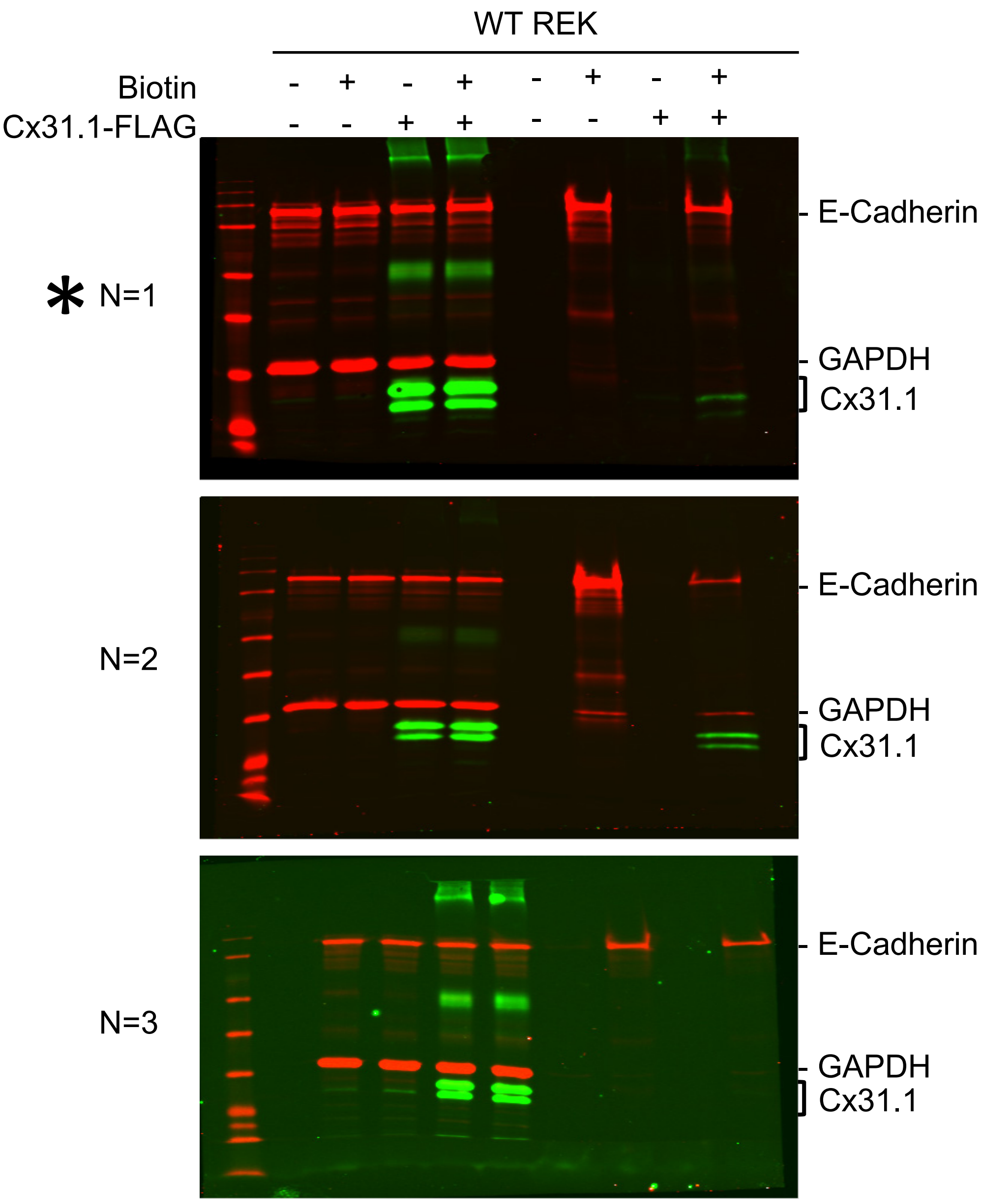

Journal of Cell Science • Supplementary information

Fig. 4 Full Blots

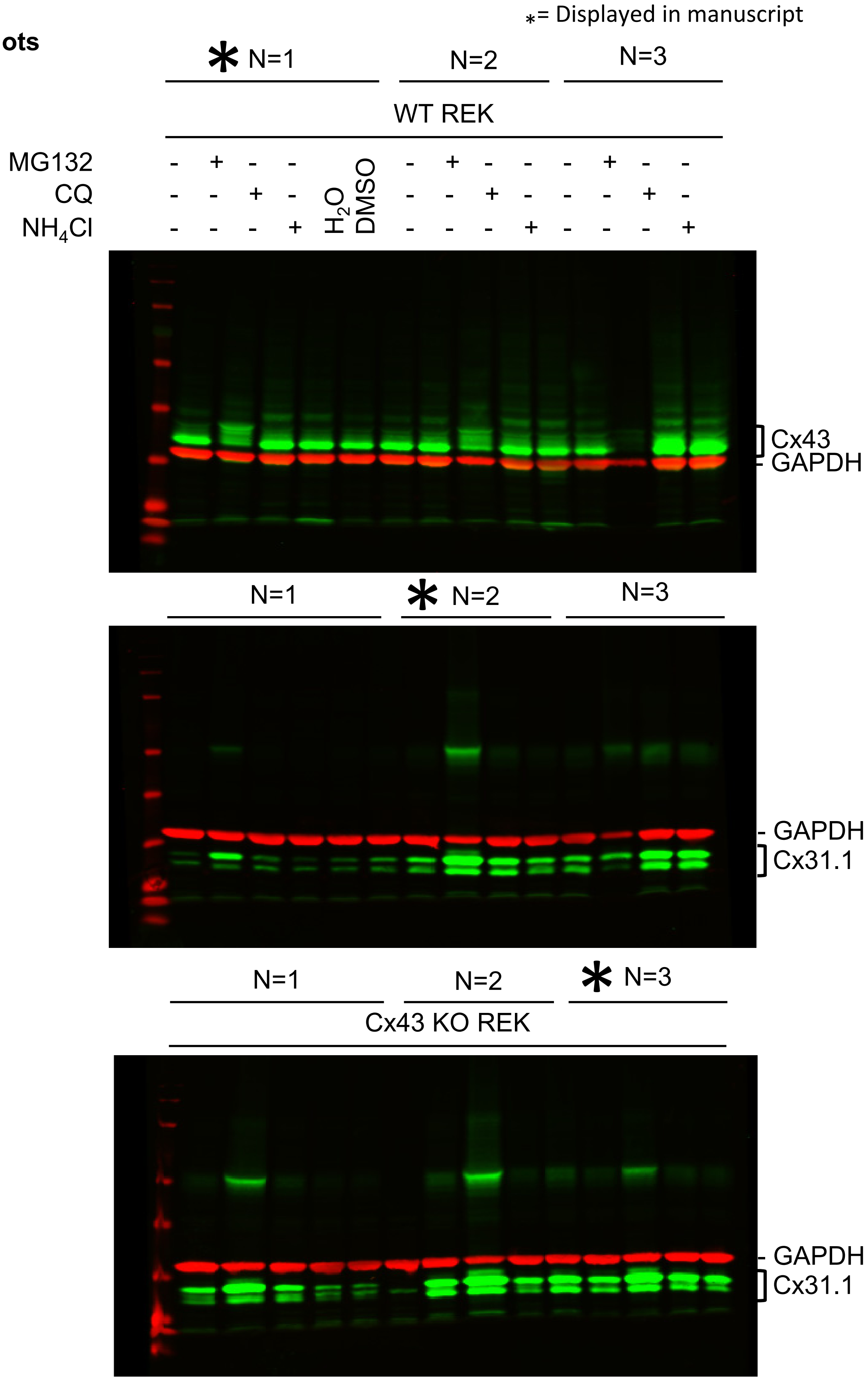

Journal of Cell Science • Supplementary information

Full blots for Fig. 5.

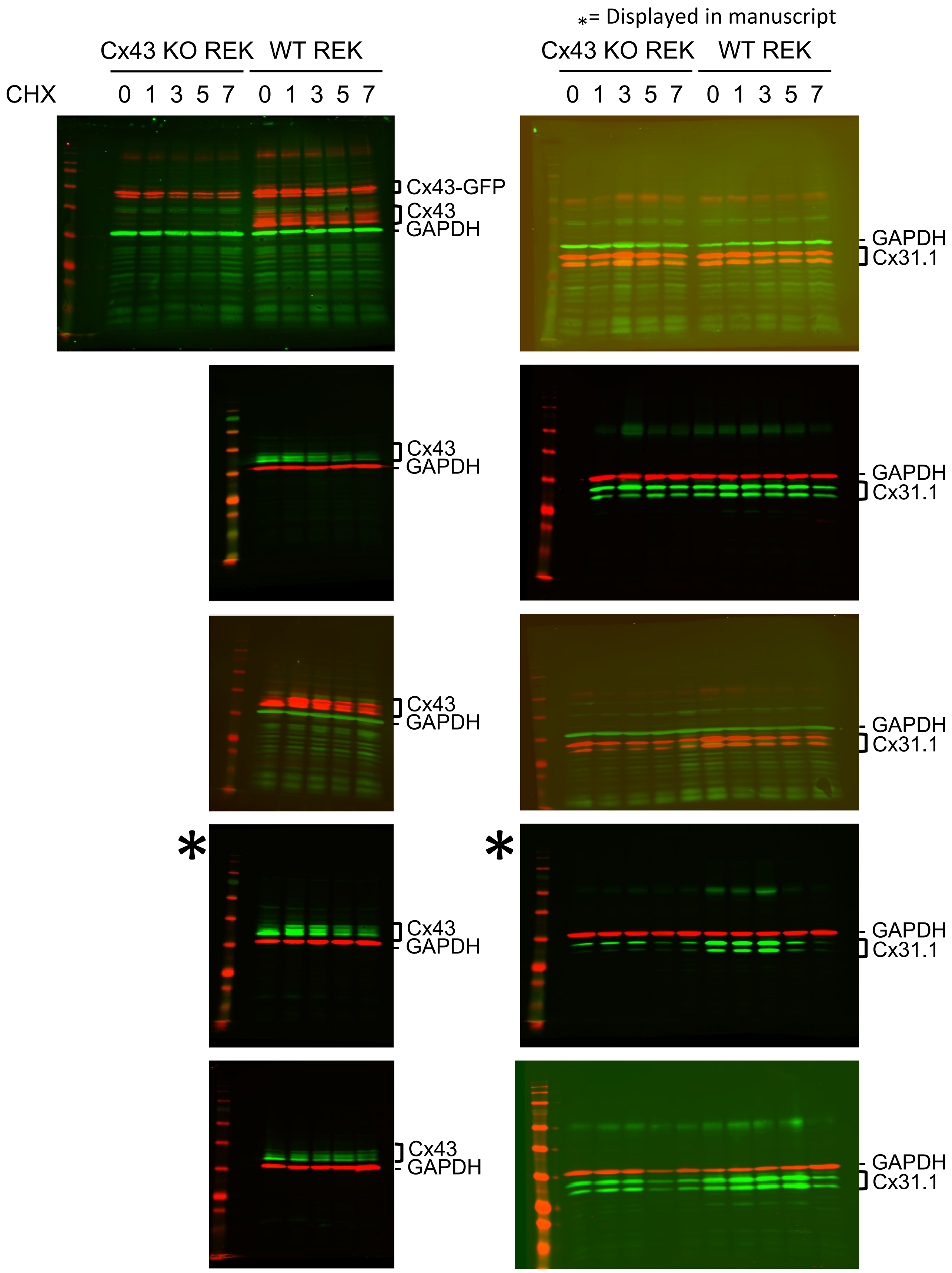

Journal of Cell Science • Supplementary information

Full blots for Fig. 5 and Fig. S6.

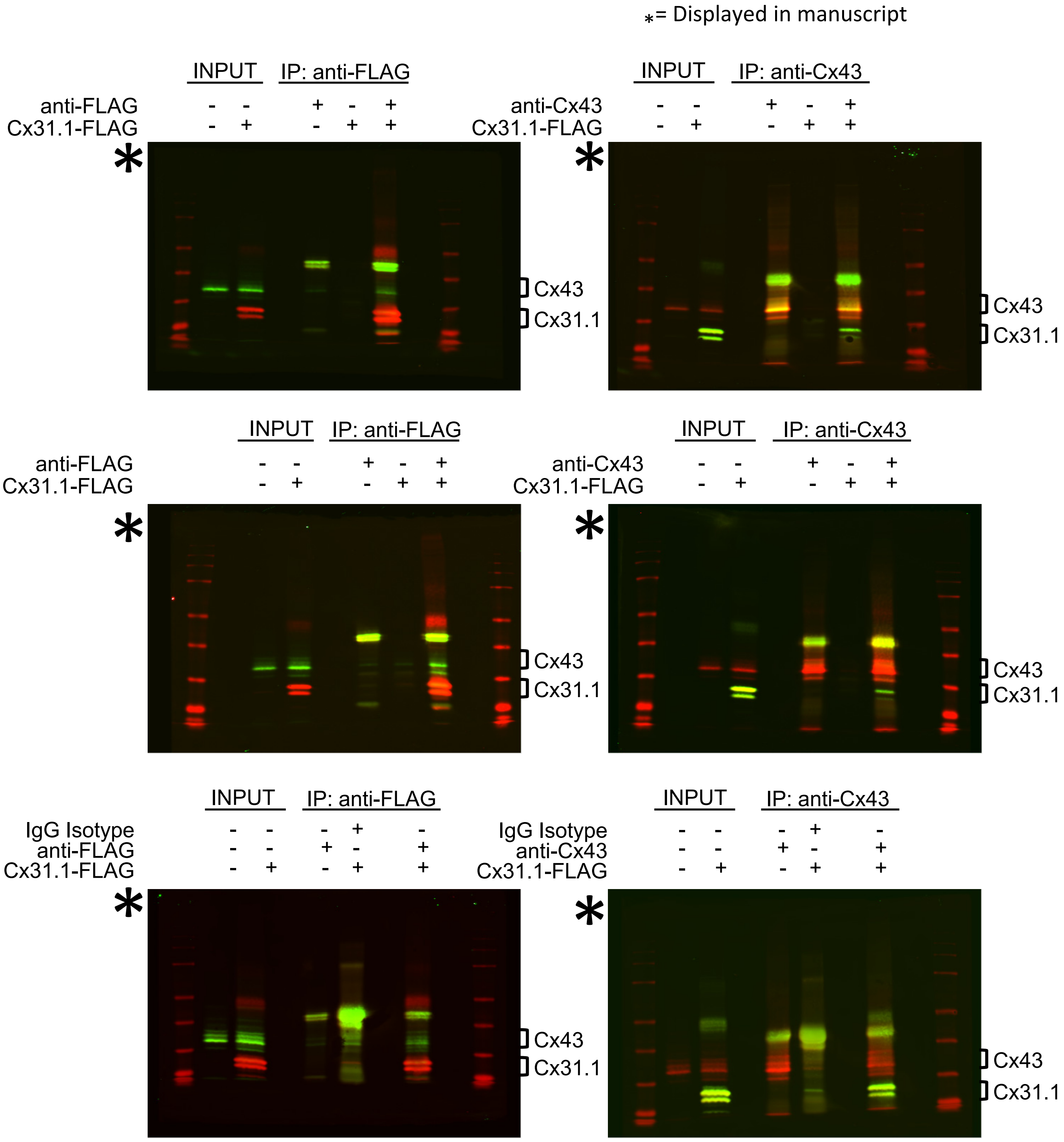

Full blots for Fig. S5.

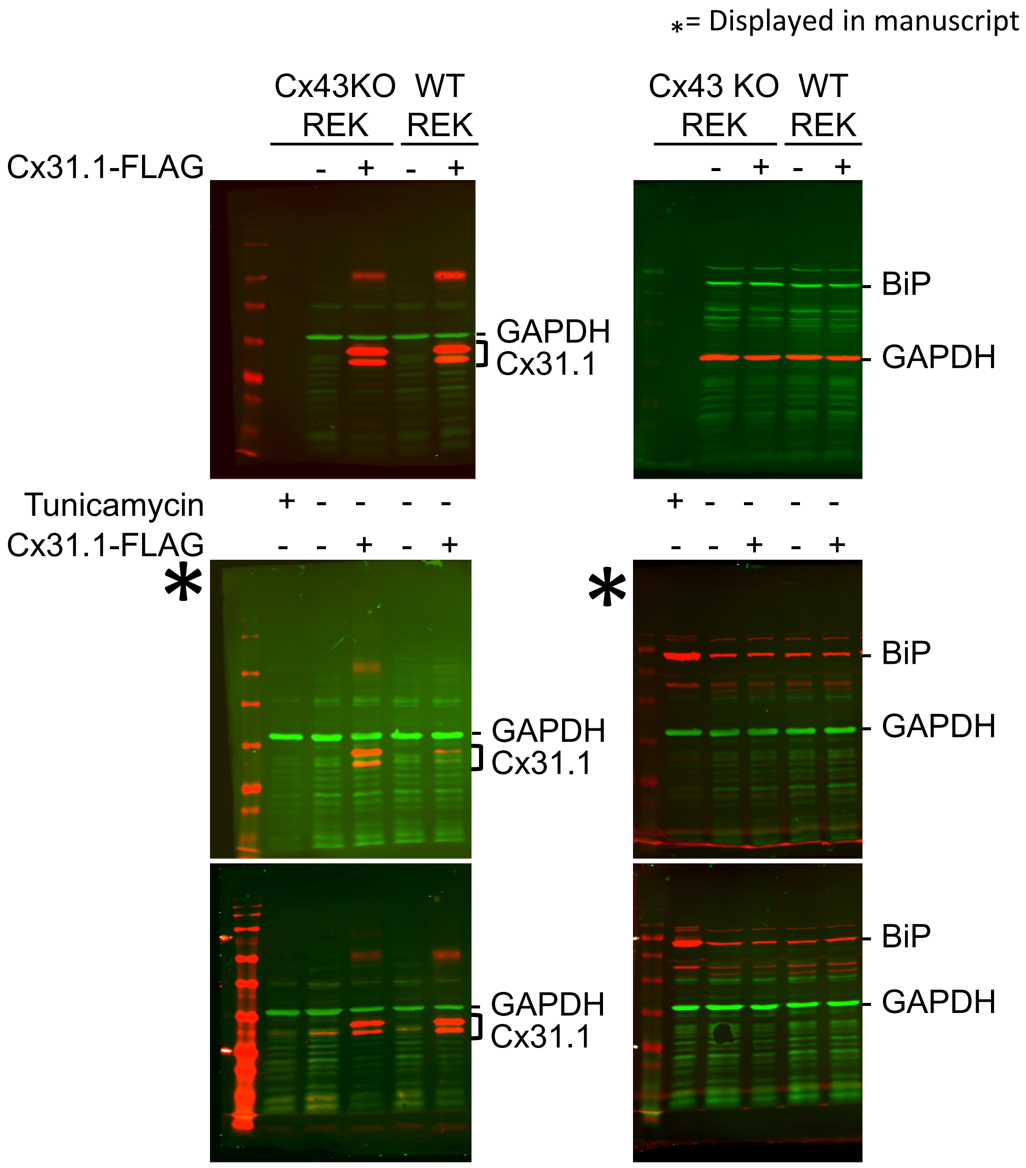

Fig. S8. Blot transparency.

Journal of Cell Science • Supplementary information
